# National Food Consumption Survey (NIPNOD 2018–2023): Results of Dietary Habits and Diet Quality Among Adolescents in Croatia

**DOI:** 10.3390/children13060799

**Published:** 2026-06-10

**Authors:** Ana Ilić, Ivana Rumbak, Martina Pavlić, Lidija Šoher, Daniela Čačić Kenjerić, Jasna Pucarin-Cvetković, Darja Sokolić

**Affiliations:** 1 Department of Nutrition, Food Quality and Safety, University of Zagreb Faculty of Food Technology and Biotechnology, Pierottijeva 6, 10000 Zagreb, Croatia; ana.ilic@pbf.unizg.hr; 2 Centre for Food Safety, Croatian Agency for Agriculture and Food, Ivana Gundulića 36b, 31000 Osijek, Croatia; martina.pavlic@hapih.hr (M.P.); darja.sokolic@hapih.hr (D.S.); 3 Department of Food and Nutrition Research, Faculty of Food Technology Osijek, Josip Juraj Strossmayer University of Osijek, Franje Kuhača 18, 31000 Osijek, Croatia; lidija.soher@ptfos.hr (L.Š.); daniela.kenjeric@ptfos.hr (D.Č.K.); 4 Department of Environmental and Occupational Health and Sports Medicine, Andrija Štampar School of Public Health, School of Medicine, University of Zagreb, Rockefeller St. 4, 10000 Zagreb, Croatia; jasna.pucarin@snz.hr; 5 Division for Environmental Health, Croatian Institute of Public Health, Rockefeller St. 7, 10000 Zagreb, Croatia

**Keywords:** adolescents, Croatia, diet quality, dietary intake, dietary survey, dietary habits, EU Menu, food sources, national survey

## Abstract

**Highlights:**

**What are the main findings?**

- Croatian adolescents’ dietary patterns showed moderate overall diet quality with imbalanced energy and macronutrient intake, identifying grains, grain products, potatoes and tubers, meat, poultry, fish, eggs, cakes, confectionery, sweets, and sugar food groups as the main sources of energy.
- A higher percentage of adolescents had irregular meal frequency, with breakfast skipping as one of the most prominent behaviors, and distinct dietary patterns marked by differences in snack consumption.

**What are the implications of the main findings?**

- The results provide an important scientific basis for developing food-based dietary guidelines for adolescents.
- The findings from this study highlight the need for targeted public health strategies to improve dietary patterns among Croatian adolescents.

**Abstract:**

**Background/Objectives**: In Croatia, national data on adolescents’ dietary habits are limited, resulting in a lack of evidence-based food-based dietary guidelines and public health interventions. This study aims to conduct an in-depth evaluation of dietary habits in a national sample of Croatian adolescents stratified by region, sex and age, from the National food consumption survey on adolescents and adults (NIPNOD 2018–2023). **Methods**: This cross-sectional study included 258 adolescents (50.4% boys; aged 10 to < 18) from the NIPNOD 2018–2023 survey (OC/EFSA/DATA/2017/01), conducted according to the EU Menu methodology. For analysis, the sample was divided into two age groups (10–13 and 14–17 years). To assess dietary intake, two 24 h recalls were analyzed using NutriCro^®^ v. 3.0 software. Dietary intake was compared with European Food Safety Authority dietary reference values (DRV). The contribution of 14 food groups to daily energy intake was analyzed. Diet quality was assessed using the Diet Quality Index for Adolescents (DQI-A). **Results**: The mean daily energy intake was 1820 ± 529 kcal, consisting of 45.5 ± 7.0% carbohydrates, 37.8 ± 6.3% fats, and 15.1 ± 3.1% protein. The observed two-day mean intake suggested that 51.6% of adolescents had carbohydrate intake within the EFSA DRV range, while 5.4% and 32.2% had protein and fat intake within the EFSA DRVs, respectively. The main contributors to daily energy intake were grains and grain products (31.5%), meat, poultry, fish, and eggs (18.1%), and cakes, confectionery, sweets, and sugar (14.9%). Frequent breakfast skipping and snack consumption were common, particularly among older adolescents. Adolescents had moderate overall diet quality (57.4 ± 11.6% DQI-A), with no differences between age groups. **Conclusions**: Analysis of the dietary habits of adolescents in Croatia indicates that most have inadequate macronutrient intake, irregular meal frequency, and moderate overall diet quality. These results highlight the need to develop public health strategies and interventions to improve dietary habits among adolescents in Croatia.

## 1. Introduction

Although there are several age cutoffs for adolescence, according to European Food Safety Authority (EFSA) guidelines for conducting standardized research in Europe, adolescence is defined as the period from 10 years to strictly under 18 years [1,2]. This turbulent period is marked by rapid growth, pubertal hormonal changes, and cognitive and psychosocial development. These factors influence both the dietary requirements and behaviors of adolescents, which may consequently affect their nutritional status [1,3]. In this age group, impaired nutritional status is already observed. The latest study from the NCD Risk Factor Collaboration, covering 1999 to 2022, found that worldwide obesity rates among children and adolescents (5–19 years) increased from 8% to 20%, while underweight rates decreased [4]. Although there is a lack of studies on representative samples of adolescents in Croatia, an increasing trend in overweight and obesity has been observed in this age group [5,6]. Moreover, an alarming finding is that in a representative sample of school-age children (6–9 years), the prevalence of overweight is 17.6% and the prevalence of obesity is 10.1% [7]. Childhood obesity increases the risk of obesity in adolescence and adulthood, leading to a higher likelihood of developing chronic non-communicable diseases during both childhood and adulthood, as well as increased mortality in adulthood [8,9].

An unhealthy diet is a major behavioral risk factor for non-communicable diseases, including overweight and obesity [10]. The HELENA study has been one of the first comprehensive European multicountry studies focused on adolescents’ diets. It was estimated that adolescents had adequate energy intake, and the proportions of energy from carbohydrates, fat, and protein were sufficient, although fat quality was low compared to DACH references [11]. Currently, among European countries, adolescents’ eating behavior differs from recommended dietary guidelines for energy and nutrient intake, as well as food consumption. Generally, consumption of fruits, vegetables, milk and dairy products, plant-based protein sources, fish, and water is low, while intake of sweetened foods and meat is excessive [12,13,14,15,16,17,18]. Moreover, there was no improvement in overall diet quality over the past two decades, during which adolescents consistently had low overall diet quality [19,20,21,22]. Indeed, in Croatia, smaller studies among adolescents indicate low adherence to recommendations for dietary intake and food group consumption [5,23,24,25,26,27]. Additionally, indicators of poor eating habits among Croatian adolescents include low adherence to the Mediterranean diet, skipping meals, and increased evening food consumption [6,24,28].

Adolescents’ eating habits are influenced more by their emotional and psychosocial needs and knowledge than by the home environment. Socioeconomic status and food availability may also affect their food choices. However, they often experience conflict among their nutritional knowledge, peer pressure, and body image. Their strong need for independence and decision-making is also reflected in their food choices [1,3]. It is well known that eating habits formed in childhood and adolescence often persist into adulthood [29]. As abstract thinking develops and awareness of the importance of proper nutrition for long-term health increases, targeted nutritional interventions can lead to positive changes in eating behavior [3,30]. To enable targeted public health interventions and improve public understanding of nutritional messages, the World Health Organization (WHO) and the Food and Agriculture Organization, within the framework of the European Food and Nutrition Action Plan, encourage the development of food-based dietary guidelines (FBDG) for different population groups to address their nutritional needs. However, these guidelines should be based on the current dietary behavior of specific populations, as they provide information in simple, understandable language for the general public about proper nutrition and emphasize necessary changes that currently negatively affect health outcomes [10,31]. In 2022, it is estimated that there were 92 FBDGs intended for infants under 24 months, children, and adolescents, of which 44.5% were specific guidelines, and the rest were incorporated into general population FBDGs [32]. In Croatia, food guidelines exist for children in grades 1 to 4 and grades 5 to 8 of primary school [33,34]. These guidelines were developed as part of the public health project “Živjeti zdravo”, implemented by the Croatian Institute of Public Health, with their development being one of the project’s tasks. Although these guidelines are not FBDGs in the classical sense, as they were not created according to WHO methodology, they are publicly available and used in Croatia.

The aim of this study is to provide a comprehensive assessment of dietary habits in a sample of Croatian adolescents aged 10 to 17 years, using data from the National food consumption survey on adolescents and adults from 10 to 99 years of age (NIPNOD 2018–2023). This includes assessment of dietary intake, comparison with EFSA dietary reference values (DRV), and identification of the main food sources of public health importance. In addition, the study aims to estimate overall diet quality using the Diet Quality Index for Adolescents (DQI-A) and to assess meal frequency. The results should provide an updated scientific basis for developing evidence-based food-based dietary guidelines (FBDG) for adolescents, inform public health policies, guide targeted nutritional interventions, and support further development of strategies to improve adolescent nutrition in Croatia.

## 2. Materials and Methods

### 2.1. Methodological Approach and Ethical Aspects

For the purpose of this cross-sectional study, data from 258 adolescents (aged 10 to <18 years) were drawn and analyzed from the NIPNOD, conducted from January 2018 to June 2023. Socio-demographic, lifestyle, anthropometric and dietary data were collected using the EU Menu methodology [2] and are described in detail in the report [35]. Before data collection, the study protocols underwent ethical review and were approved by the Ethics Committee of the Institute for Medical Research and Occupational Health (Class: 01-18/20-02-2/1; Reg. No: 100-21/20-18) and were carried out in accordance with the ethical principles of the Declaration of Helsinki.

### 2.2. Sample Size and Recruitment Protocol

This cohort represents a sample of adolescents in Croatia. The sample size was determined using age and sex distribution from the Croatian Statistical Bureau [36], resulting in a total sample of 261 participants. This overall sample size complies with EFSA guidelines for minimum participant numbers in the targeted population surveys [2]. The participants were recruited using a stratified random sampling approach from the Ministry of Internal Affairs database [35]. In the first step of recruitment, the population was stratified into six regions proportional to the population size of each region. Participants were then randomly selected within each regional stratum according to sex and age. Participants were excluded if they were hospitalized or residing in collective households. Adolescents with specialized diets (e.g., allergy, coeliac diet, vegetarian diet, etc.) were not excluded from the present study. The eligible sample included 1182 adolescents, of whom 879 were not contactable, and 42 refused to participate in the survey. Additionally, 3 adolescents did not complete the second 24 h recall and were excluded from the final data analysis. The final dataset consisted of 258 adolescents (21.8% participation rate), with 130 boys and 128 girls.

For each participant, a parent or guardian signed a consent form after being informed of the survey’s aims and protocols. Participation in the research was voluntary, and participants were informed of their right to withdraw consent at any time during or after data collection.

### 2.3. Socio-Demographic and Lifestyle Data Collection

Data on age, sex, and region were collected during the recruitment of adolescents, while informations on adolescents’ education and employment, parental education and employment, household characteristics (size and income), health-related data (presence of choric disease: yeso/no, specification of chronic disease, food and non-food allergies) and dietary behavior (frequency of meal consumption during one-week, special diet, use of supplements) were collected using a general questionnaire through in-person or video call interviews.

Physical activity level was assessed using the short form International Physical Activity Questionnaire, which reflects participants’ physical activity over the past week [37,38]. Each participant was classified into one of three physical activity levels: low, moderate, or high. Classification followed standard IPAQ scoring criteria based on the frequency and duration of activity, as well as MET values for walking, moderate-intensity activity, and vigorous-intensity activity. Total MET-minutes per week were calculated as the sum of weekly MET-minutes for walking, moderate, and vigorous activities.

### 2.4. Anthropometric Characteristics

Anthropometric data were collected by trained interviewers following standard protocols [39]. A digital scale (Seca, type 877, Vogel and Halke GmbH and Co., Hamburg, Germany) was used to measure body weight to within ±0.1 kg, and a portable stadiometer (Seca, type 217, Vogel and Halke GmbH and Co., Hamburg, Germany) was used to measure height to within ±0.1 cm. Body mass index (BMI) was calculated for each participant based on these measurements and used as a nutritional status indicator. The WHO cut-off for BMI-for-age was used to categorize adolescents by nutritional status [40]. Height- and BMI-for-age z scores were generated using WHO AnthroPlus software version 1.0.4. [41].

### 2.5. Assessments of Dietary Intake and Food Groups

Dietary intake and food group consumption were assessed using two 24 h recalls on non-consecutive days. The scheduling algorithm for the 24 h recalls ensured data collection for each day of the week across all four seasons at the population level. Trained interviewers collected the 24 h recalls from adolescents in the presence of their parents or guardians. All data, including cooking methods, recipes, product brands, supplement use, and amounts consumed, were collected through interviews and entered into the NutriCro^®^ v. 3.0 software [35]. Portion sizes were estimated using country-specific and PANCAKE picture books [42,43,44], serving sizes from food packaging, or predefined household measures. All recipes were broken down into individual food items. To provide information on dietary intake, NutriCro^®^ v. 3.0 software includes the Croatian food composition tables [45], the Danish Food Composition Database [46], and information from nutrition labels of specific food products and supplements from the Croatian market that were not present in either database. To ensure data accuracy, the software also incorporates food weight yield and retention factors [47]. The estimated energy and macronutrient intake were compared to the EFSA DRV [48]. Although foods and beverages in the program were classified according to the FoodEx2 classification, for analysis, all foods and beverages were divided into 14 categories (Supplementary Table S1). The proportion of the amount consumed and the contribution to daily energy intake were calculated for each food group.

### 2.6. Diet Quality Index for Adolescents

The diet quality of the participants was assessed using the DQI-A, which has been validated in adolescents from several European countries in the HELENA study by Vyncke et al. (2012) [49]. The DQI-A was used in its original form and was not modified for the purpose of this study. The DQI-A included three components: dietary quality, dietary diversity, and dietary equilibrium. The average of these three components represented the final score, which can range from −33% to 100%, with a higher score indicating better diet quality.

For each participant, all foods and beverages reported in the two 24 h recalls were classified into eleven predefined DQI-A food groups according to the methodology: (1) water, (2) bread and cereals, (3) potatoes and grains, (4) vegetables, (5) fruits, (6) milk products, (7) cheese, (8) meat, fish, and substitutes, (9) fats and oils, (10) snacks and candy, and (11) sweetened drinks and fruit juices. The first nine food groups were recommended, while the last two were not. Each food group also has maximum and minimum consumption recommendations based on the Flemish FBDG [50]. The amounts of food and beverages reported in 24 h recalls were expressed in grams or milliliters, consistent with the Flemish FBDG. The classification and scoring procedures followed the original DQI-A protocol.

Afterward, each of the three index components was calculated. Dietary quality reflected the optimal choice of foods from each food group according to the preferences in the Flemish food-based dietary guidelines. It was calculated by multiplying the portion size of each food group by a factor: 1—“preference” foods, 0—“intermediate” foods, and −1—“low-nutrient, energy-dense” foods. The obtained value for each food group was summed, divided by the total amount of food eaten in one day, and multiplied by 100. The possible score range in this component was from −100% to 100%. Dietary diversity was calculated as the percentage of nine recommended groups in which participants consumed at least one minimum portion. This component can range from 0 to 100%. Dietary equilibrium reflected how the consumed portion size aligns with the Flemish FBDG. It was calculated as the difference between the adequacy score percentage of the minimum recommended intake for each main food group and the percentage of intake exceeding the upper recommended level. In the adequacy score, values greater than 1 were truncated to 1, while in the exceeding score, values greater than 1 were truncated to 1 and values less than 0 were truncated to 0. This component may also range from 0 to 100%.

### 2.7. Data Handling and Analysis

Data were processed, analyzed, and visualized using IBM SPSS Statistics (version 23.0; IBM Corp., Armonk, NY, USA) and Microsoft Excel 2016 (version 16.0.5413.1000; Microsoft Corporation, Redmond, WA, USA). All data were presented as a total sample of adolescents and for two age groups: 10–13 years and 14–17 years. All variables were fully observed except for frequency of meal consumption, for which mean value imputation was used due to the low rate of missing data (*n* = 1 for breakfast, morning snack, lunch, and dinner; *n* = 2 for afternoon snack; *n* = 3 for evening snack). Under and over-reports were identified using established protocols based on Goldberg et al. [51] and Black et al. [52], as it is detailed in report [35]. These participants were not excluded from the primary analysis, but a post hoc sensitive analysis was conducted, including only plausible reporters. No survey weights were used. Socio-demographic, lifestyle, and anthropometric characteristics are presented as mean and standard deviation for continuous variables and as percentages for categorical variables. Dietary data are calculated as observed individual means [53]. Energy and macronutrient intake are presented as mean and standard deviation, as well as the 5th, 25th, 50th, 75th, and 95th percentiles. The distributions of adolescents by observed macronutrient intakes relative to EFSA DRV are shown as percentages. The consumption of food groups is presented as the mean value of their contribution to overall food intake and daily energy intake. Meal consumption frequency is defined as the proportion of children who consume a specific meal (breakfast, morning snack, lunch, afternoon snack, dinner, and evening snack) within a week. Within each age group, adolescents were divided into two groups using K-means cluster analysis. A two-cluster solution was selected based on the agglomeration schedule results from hierarchical cluster analysis using Squared Euclidean distance and Ward’s method. The DQI-A is presented as the mean and standard deviation. Differences in categorical variables between the two adolescent age groups were tested using the Chi-square test or Fisher’s exact test. Differences in continuous variables were tested using the *t*-test or the Mann–Whitney U test, depending on the results of the Shapiro–Wilk test for normality. The threshold for statistical significance was set at *p* < 0.05.

## 3. Results

The study sample comprised 14.3 ± 3.2-year-old adolescents (*n* = 258; 50.4% boys and 49.6% girls). Of the total sample, 47.7% were in the 10 to 13 age group, while the remainder were in the 14 to 17 age group. Only 10 participants in the age group 14 to 17 years collected data independently, while parents collected data for the other adolescents. Both adolescent age groups were similar in their socio-demographic and lifestyle characteristics (Table 1). Most adolescents were from Zagreb and Northern Croatia and primarily lived in urban areas. Most of their parents had higher levels of education, were employed for pay or profit, and belonged to a higher income category. The majority of adolescents were healthy, but nearly 50% had a low level of physical activity.

Anthropometric measurement analysis (Table 2) shows that the average z-score for BMI-for-age was 0.2 ± 1.1. Nearly one quarter of adolescents were overweight, and 4.7% were obese. No difference in weight status was found between adolescent age groups.

In the total sample (Table 3), the mean daily energy intake among adolescents was 1820 ± 529 kcal, with no differences between age groups. Plausible energy intake was observed in 67.4% of adolescents (75.8% in the 10–13 age group and 60.7% in the 14–17 age group).

**Figure 1:**
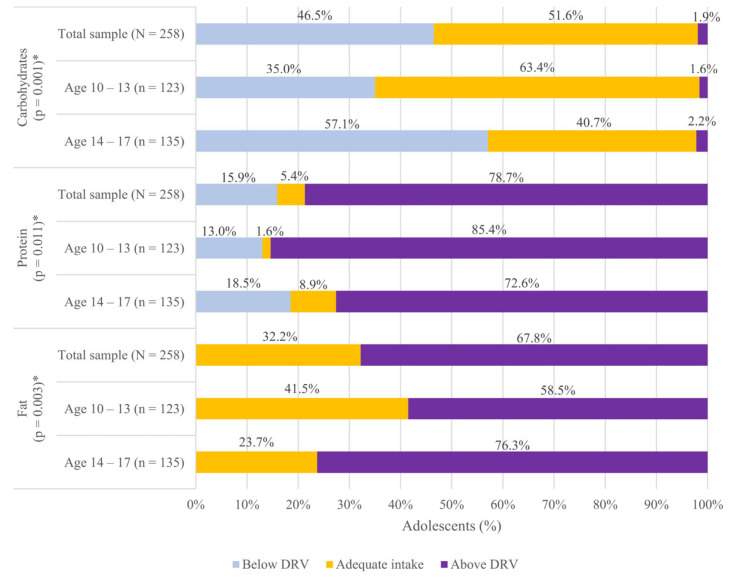
Distribution of adolescents from the NIPNOD 2018–2023 survey by macronutrient intake relative to EFSA DRVs. * Differences between age groups were tested using the Chi-square or Fisher’s exact test (*p* < 0.05).

Regarding carbohydrate intake, differences were observed only in the contribution of carbohydrates to daily energy intake, with the younger age group having a higher contribution (47.3 ± 6.4% kJ vs. 43.9 ± 7.1% kJ; *p* < 0.001) compared to the older age group. Based on the mean of two 24 h recalls, a higher observed proportion (*p* = 0.001) of adolescents in the younger age group (63.4%) had carbohydrate intake within the EFSA DRV range compared to the older age group (40.7%) (Figure 1).

For protein intake, the older age group had a significantly higher total protein intake than the younger group (72.2 ± 27.0 g vs. 64.4 ± 18.5 g; *p* = 0.021). However, when expressed per kilogram of body weight, adolescents in the older group had a lower protein intake (1.2 ± 0.5 g/kg BW vs. 1.4 ± 0.5 g/kg BW; *p* < 0.001). In the total sample (Figure 1), 15.9% of adolescents had protein intake below the EFSA DRV thresholds, while 78.7% had intake above these thresholds based on observed two-day mean intake. Additionally, reflecting the observed two-day mean intake, a higher observed proportion of adolescents in the younger age group (85.4%) had intakes above the recommended level compared to those in the older age group (72.6%) (*p* = 0.011).

Fat intake as total amount (82.0 ± 32.0 g vs. 71.7 ± 22.9 g; *p* = 0.006) and as a share of energy (39.0 ± 6.1% kJ vs. 36.5 ± 6.1% kJ; *p* = 0.001) was higher in the older age group. No adolescent had observed a two-day mean fat intake below the EFSA DRV recommended range (Figure 1), while a considerable proportion had an intake above the recommended range. This pattern was more pronounced in the older age group (76.3% vs. 58.5%; *p* = 0.003). The Supplementary Material provides detailed information on mean daily energy and macronutrient intake (Tables S2 and S3), as well as the distribution of adolescents according to EFSA DRV by sex and age group (Table S4).

**Figure 2:**
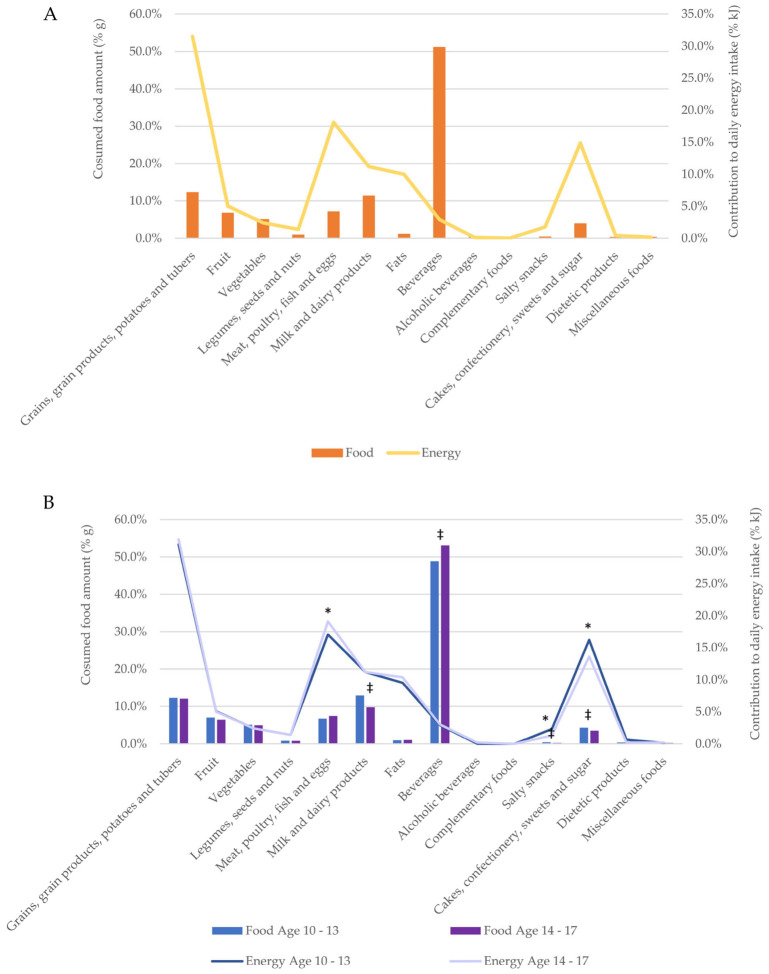
Relative contribution of food groups to total daily food consumption and daily energy intake in adolescents from the NIPNOD 2018–2023 survey. (**A**) Total study sample; (**B**) differences between age groups. Differences (‡ contribution to daily food consumption; * contribution to daily energy intake) between age groups were tested using the independent *t*-test or Mann–Whitney U test (*p* < 0.05).

The results of the analysis of food group consumption (g/day) are presented in Supplementary Table S5, with sex-specific analyses in Tables S6 and S7. The relative contributions of food groups to total daily food consumption and daily energy intake are shown in Figure 2 and detailed in Tables S8 and S9. The analysis revealed that the three most consumed foods were beverages (51.1%), grains, grain products, potatoes and tubers (12.2%), and milk and dairy products (11.3%). The three food groups contributing most to daily energy intake were grains, grain products, potatoes and tubers (31.5%), meat, poultry, fish and eggs (18.1%), and cakes, confectionery, sweets and sugar (14.9%). Differences between age groups were found in the relative contribution of four food groups to total food consumption. Younger adolescents had a higher relative intake of milk and dairy products (12.9 ± 8.9% g vs. 9.8 ± 7.1% g; *p* = 0.006), cakes, confectionery, sweets and sugar (4.3 ± 0.9% g vs. 3.5 ± 3.9% g; *p* = 0.005), and salty snacks (0.4 ± 0.9% g vs. 0.2 ± 0.7% g; *p* = 0.034), while older adolescents had a higher relative intake of beverages (53.1 ± 14.5% g vs. 48.9 ± 13.4% g; *p* = 0.011). Although differences were observed in the consumption of milk and dairy products and beverages, their contributions to daily energy intake were similar among age groups. Differences in the contribution of cakes, confectionery, sweets, and sugar to daily energy intake (16.2 ± 10.8% kJ vs. 13.6 ± 12.1% kJ; *p* = 0.020) and salty snacks (2.3 ± 4.8% kJ vs. 1.3 ± 4.1% kJ; *p* = 0.034) followed the same pattern as the observed differences in the amounts of food consumed from these groups. In contrast, older adolescents had a significantly higher (*p* = 0.048) contribution to daily energy intake from the meat, poultry, fish, and egg food group (19.1 ± 8.6% kJ) than younger adolescents (17.1 ± 7.8% kJ), although no difference was observed in the amount of food consumed. Data on the contributions of individual food groups to macronutrient intake are shown in Supplementary Table S10.

**Figure 3:**
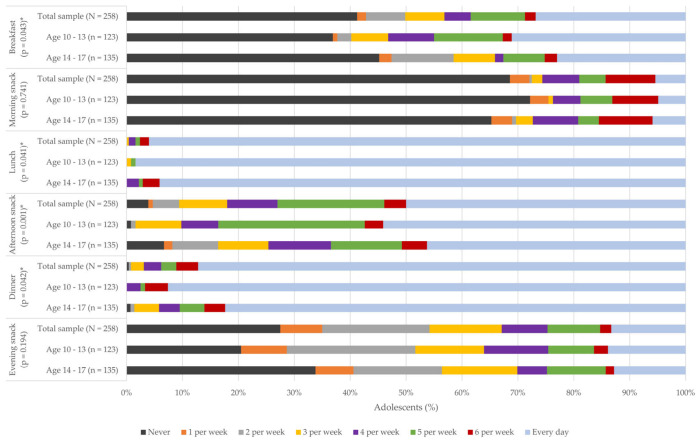
Distribution of adolescents from the NIPNOD 2018–2023 survey by frequency of meal consumption. * Differences between age groups were tested using the Chi-square or Fisher’s exact test (*p* < 0.05).

**Figure 4:**
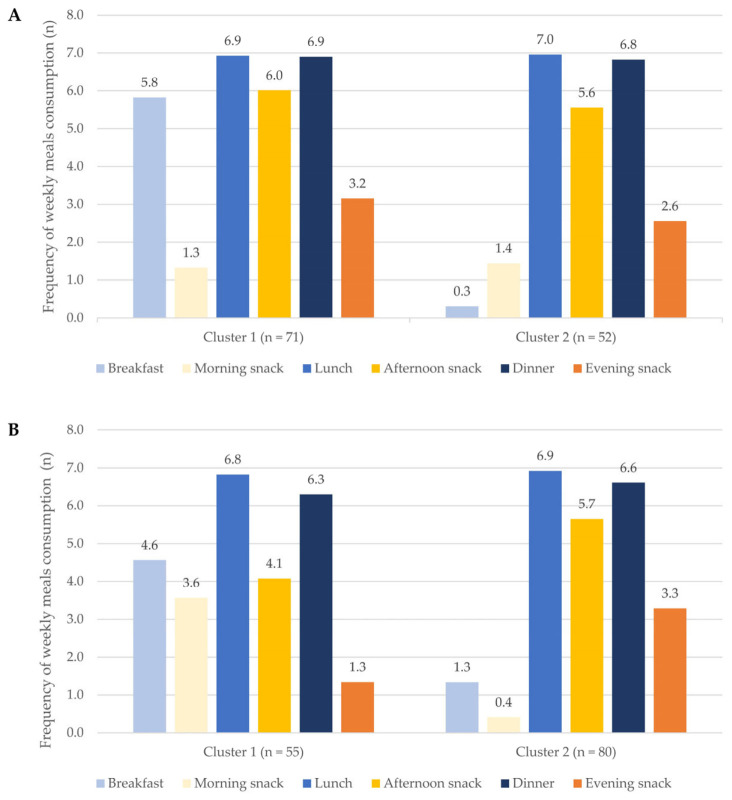
Cluster profiles of weekly meal consumption frequencies among adolescents aged 10–13 years (**A**) and 14–17 years (**B**) from the NIPNOD 2018–2023 survey.

The weekly frequency of meal consumption and cluster analysis-based consumption patterns are shown in Figure 3 (detailed Supplementary Materials Table S11) and Figure 4, respectively. Regarding breakfast skipping, 41.2% of adolescents reported never eating breakfast. Additionally, a higher proportion of breakfast skipping was observed in the older age group (*p* = 0.043). Lunch and dinner were the most frequently consumed meals among adolescents, with younger adolescents reporting more frequent consumption of both meals (lunch: *p* = 0.041; dinner: *p* = 0.042). Regarding snacks, a higher proportion of adolescents skipped the morning snack, while the afternoon snack was consumed more frequently. A higher frequency of afternoon snack consumption (*p* = 0.001) was observed among younger adolescents. About one quarter of adolescents in both age groups never consumed an evening snack. According to the K-means cluster analysis, two main clusters were identified in both age groups. Among adolescents aged 10–13 years, clusters differed in breakfast consumption frequency, while other meals were consumed at similar frequencies. In this age group, 57.7% were in cluster 1, characterized by more frequent breakfast consumption. Among adolescents aged 14–17 years, cluster 1 (40.7%) was characterized by more frequent consumption of breakfast and morning snacks and less frequent consumption of afternoon and evening snacks. In contrast, cluster 2 (59.3%) was characterized by irregular dietary patterns, including breakfast skipping and more frequent evening snack consumption.

According to the results presented in Table 4 and in detail by sex in Table S12, the mean DQI-A score was 57.4 ± 11.6% in the total study sample. No difference in diet quality was observed between adolescent age groups. Additionally, no differences were found in the individual components of the DQI-A.

## 4. Discussion

This study is the first one that provides a comprehensive overview of the dietary habits of adolescents in Croatia. Similar to adolescents across other European countries [11,12,13,14,15,16,17,18,21,22], observed intake based on the mean of two 24 h recalls suggests that a lower proportion of Croatian adolescents had macronutrient intakes within the EFSA DRVs. Their daily energy intake came mainly from grains, grain products, potatoes and tubers, meat, poultry, fish and eggs, as well as cakes, confectionery, sweets, and sugar food groups. Finally, they had moderate overall diet quality, as assessed by the DQI-A.

These findings are of concern because inadequate nutrition and low level of physical activity are key factors in the etiology of adolescent obesity. According to the Health Behaviour in School-age Children Study (HBSC), Croatian adolescents aged 11, 13, and 15 are among those in countries with the highest prevalence of overweight and obesity [54]. Indeed, in this study, 21.7% of Croatian adolescents were classified as overweight and 4.7% as obese, which is higher than reports from other European national studies [14,16,55,56]. Moreover, nearly half of adolescents from the present study had low levels of physical activity. Adolescent dietary behavior is influenced by individual, family, and environmental factors. One of the most prominent environmental factors is the school setting, as adolescents often spend up to six or more hours there. According to EFSA, adolescence refers to the period from 10 to under 18 years, which includes children in the higher grades of primary school and those in secondary school. In Croatia, primary schools are required to provide meals according to current regulations [57,58]. However, for students in higher grades, meals are offered as morning or afternoon snacks, depending on the student’s schedule. Not all schools offer snack meals for students in these grades, and students may choose whether to take this meal option. Since 2023, free meals have been provided for all primary school children in Croatia [59]. Vending machines are not recommended in primary schools, and only 4.2% of schools had them [60]. In contrast, adolescents attending high school do not have access to school cafeterias. Instead, they rely on fast food kiosks offering items such as sandwiches and sweets, or on vending machines. As a result, adolescents largely depend on their own choices from shops and bakeries located near schools.

Adolescence is a period of rapid growth and hormonal changes that affect the nutritional needs of this population [3]. Energy requirements must align with these changes and the increased need for physical activity [61]. EFSA established recommendations for energy intake based on the sex, age, and physical activity level (PAL) of adolescents, ranging from 1818 kcal (girls, 10 years, PAL 1.6) to 3675 kcal (boys, 17 years, PAL 2.0) [48]. According to European national surveys published up to June 2016, the mean energy intake for girls aged 10 years and older was 7.7 MJ (approximately 1840.34 kcal), and for boys, 9.4 MJ (approximately 2246.65 kcal) [17]. In this study sample, adolescents had a mean daily energy intake of 1820 ± 529 kcal, with some differences observed between boys and girls (Tables S2 and S3). This energy intake is similar to that reported in other European countries, with boys showing higher intakes than girls [13,16,18,62,63,64,65,66,67]. Only adolescents from Italy and the Netherlands have reported higher energy intakes than those observed in Croatia [14,55]. The EU Menu methodology provided guidelines on how to detect energy misreporting in the national surveys, accounting for basal metabolic rate and physical activity level [2]. In Croatia, 32.6% of adolescents were classified as under-reporters, while the others were considered plausible reporters. No adolescents were classified as over-reporters. A similar pattern of a higher proportion of under-reporters and few or no over-reporters has been observed in other European countries [55,64,66,67], with the exception of Cyprus and Spain, where a lower proportion of under-reporting was observed [16]. According to the literature, a higher rate of misreporting was common during adolescence. Moreover, higher odds of under-reporting among adolescents have been associated with female sex, higher BMI, body weight concerns, higher physical activity level, skipping breakfast, and a greater relative contribution of carbohydrate and protein to daily energy intake [68,69]. In our study sample, 31.4% of adolescents had a high activity level, 21.7% were overweight, 4.7% were obese, and 41.2% skipped breakfast. These characteristics may have contributed to the higher rate of under-reporting. However, associations between these factors and misreporting were not assessed in this study. Countries may choose to include or exclude misreports in data analysis, but most studies include them [14,16,62,64,65]. The inclusion of misreports may affect the estimation of nutrient intake and food group consumption, potentially limiting the comparability of dietary data between countries. The Supplemental Materials include a post hoc analysis comparing adolescents according to the plausibility of energy intake (Tables S13–S20). In contrast to the total sample, daily energy intake differed among plausible adolescents across adolescent age groups. Other data related to daily macronutrient intake were consistent across both datasets. Furthermore, no changes were observed in the distributions of adolescents who fall within the EFSA DRV in the plausible subsample of adolescents.

The Croatian sample of adolescents did not differ from other European populations in terms of food group consumption (Supplementary Table S5) and their contribution to the daily energy intake [18,63,64,65,66,67]. Despite differences in food group classification methodology, the overall pattern indicates that the main sources of energy were grains, grain products, potatoes, and tubers, followed by meat, poultry, fish, eggs, cakes, confectionery, sweets, and sugars. Including misreports in our analysis could affect the estimation of food group consumption (Table S15). However, when examining the contribution of food groups to total food consumption (Table S16) and daily energy intake (Table S17), the only change observed was that the contribution of cakes, confectionery, sweets, and sugar no longer differed significantly between age groups in the subsample of plausible reporters.

Of the macronutrients, carbohydrate (45.5 ± 7.0% kJ) mostly contributed to the daily energy intake, followed by fat (37.8 ± 6.3% kJ) and protein (15.1 ± 3.1% kJ). According to the EFSA DRVs, carbohydrates are recommended to provide 45–60% of daily energy intake [48]. In this study, the mean contribution of carbohydrates to daily energy intake was at the lower limit of the recommended range. Moreover, 35.0% of younger adolescents and 57.1% of older adolescents had carbohydrate intakes outside the recommended range based on observed intake levels from two 24 h recalls. Similar patterns have been reported in other European countries, where carbohydrates provide less than 50% of daily energy intake and approach the lower limit of the recommended range [13,14,16,18,63,64,65,66,67]. Furthermore, about 40% of adolescents did not meet the required carbohydrate intake [16,18], with a lower proportion observed in Portugal and Spain [13,63] and a higher proportion reported in Belgium (60%) [67]. The main sources of carbohydrates in our study sample were grains, grain products, potatoes and tubers and cakes, confectionery, sweets and sugar food groups, which accounted for over 70% of carbohydrate intake (Supplementary Table S10). At the same time, these food groups were the second and seventh most consumed food groups. These results are consistent with available national European studies [65,66,67]. These findings suggest a need to encourage increased consumption of grains, grain products, potatoes, and tubers, especially whole grains, fruits, and legumes as primary sources of carbohydrates, while reducing consumption of cakes, confectionery, sweets and sugar and sweetened beverages. Increased consumption of these groups may also contribute to improving dietary fiber intake. Although dietary fiber intake was not assessed in this study, European studies have shown that a large proportion of adolescents do not meet recommended fiber intake levels [14,16,18,66].

In the total study sample, protein contributed 15.1 ± 3.1% kJ to daily energy intake. Interestingly, total protein intake was higher in the older age group, but younger adults had higher protein intake relative to body mass. When comparing observed intake with EFSA DRVs, results suggest that a large proportion of adolescents in both age groups had protein intake above the reference value, ranging from 0.91 g/kg BW in younger adolescents to 0.67 g/kg BW in older adolescents [48]. However, these results should be interpreted with caution, as adherence to EFSA DRV was assessed using two 24 h recalls. A similar high protein intake has been reported among adolescents across European countries [13,14,16,18,55,62,63,64,65,66,67]. Although higher protein intake has been considered a factor associated with obesity development during adolescence, there is currently no consensus on the limit for high protein intake [70,71,72]. The main dietary sources of protein (Supplementary Table S10) indicated that 59.8% came from meat, poultry, fish, eggs, and milk and dairy food groups, while 23.4% came from grain, grain products, potatoes, and tubers. Legumes were consumed in very small quantities and contribute minimally to daily protein intake. Only two adolescents did not consume food items from the meat, poultry, fish, and eggs food group. Additional analysis indicates that older adolescents consumed more fish products and less frozen breaded meat products than younger adolescents. Protein supplement use was observed only in a sub-sample of Croatian adolescents aged 14–17 years and was not reported among younger adolescents from this study nor among school-aged children in a previous study [7]. The use of protein supplements during adolescence is associated with self-esteem, body image, eating disorders, and athletic performance, without knowledge of potential adverse health effects [73,74,75]. It is important to support optimal protein intake during adolescence to prevent potential harmful health outcomes [72]. Protein intake should be monitored from infancy and systematically managed through kindergarten and school nutrition programs. Clear communication of age-appropriate protein requirements and their health implications is also warranted for adolescents and their parents.

The study results on short-term fat intake indicate that more than half of the adolescents had fat intake above the EFSA DRV range (20–35% kJ) [48], with this pattern more pronounced in older adolescents. Similar trends have been observed among adolescents across European countries [13,14,16,18,55,62,63,64,65,66,67]. Furthermore, it has been observed that adolescents tend to have a higher energy contribution from saturated fatty acids, with many of them exceeding the recommended intake. In contrast, monounsaturated and polyunsaturated fatty acids intake is generally low [13,14,16,65,66,67]. In the present study, intake of saturated and unsaturated fatty acids was not estimated. However, the main contributors to total fat were meat, poultry, eggs, and fish (28.8%), and fats and oils (26.4%), followed by cakes, confectionery, sweets and sugar (15.5%), and milk and dairy products (14.3%) (Supplementary Table S10). These food groups, except for fats and oils, are sources of saturated fatty acids. Within the fats and oils group, only 9.5% came from animal fats such as lard or butter, while the remainder consisted of plant-based oils, most commonly sunflower and olive oil.

Interestingly, the HELENA study showed that adolescents in Europe have an adequate intake of most nutrients [11], whereas the present study and other more recent national studies have found an imbalance in energy and macronutrient intake [13,14,16,18,55,62,63,64,65,66,67]. These differences may be partly explained by the use of different dietary reference values and methodological approaches for assessing the adequacy of energy and nutrient intake. Moreover, the discrepancy may result from assessing nutrient intake as short-term intake from two 24 h recalls or using a statistical modeling method that reflects usual intake. Such discrepancies limit comparisons between countries and may hinder the identification of dietary behaviors that could improve adherence to recommendations.

When discussing dietary patterns, it is necessary to consider the consumption of food groups. In particular, inadequate intake of fruits and vegetables has been observed among adolescents in many European countries, as well as in North America and Oceania [76]. In the present study, adolescents consumed 259.0 ± 183.2 g of fruit and vegetables per day, which is below the WHO recommendation of 400 g daily for health maintenance [77]. Definitions of fruits and vegetables vary across studies, with some including potatoes and legumes as vegetables, nuts as fruits, and 100% fruit and vegetable juices [78]. These differences in definitions may lead to variation in the estimated intake of fruits and vegetables among adolescents. In the present study, vegetables did not include legumes or potatoes, and fruits did not include nuts. Both groups included fresh, cooked, canned, or dried fruits or vegetables, as well as 100% fruit or vegetable juices. Adolescents consumed more fruits (150.3 ± 151.2 g/day; min 0.0 g/day; max. 836.3 g/day) than vegetables (108.7 ± 79.6 g/day; min 0.0 g/day; max. 420.3 g/day), with no differences observed between adolescent age groups. In the total sample, only two adolescents (0.8%) did not consume vegetables, while 57 (22.1%) did not consume fruits. Of total fruit intake, 100% fruit juices accounted for approximately 20.5 ± 56.6 mL per day (minimum 0.0 mL per day, maximum 325.0 mL per day). Fruit juice consumption in our study sample was within the American Academy of Pediatrics recommendation for adolescents (up to eight ounces, or one cup of fruit juice daily) [79]. These patterns may result from eating behaviors, as adolescents in Croatia typically eat lunch and dinner, and traditional cooked meals that include a variety of vegetables are still valued [80]. Evidence suggests that nutritional interventions can increase fruit and vegetable intake in adolescents [81]. A recent study in Croatia indicates that the school environment provides a strong foundation for implementing multi-component nutritional education among primary school children [82], which could be adapted and replicated for young adolescents who are still in the primary school environment [83]. Since 2023, younger adolescents have had access to one free school meal per day [59], and consuming school meals has been associated with increased intake of certain food groups, including fruits and vegetables [84]. However, a study conducted in primary schools in the city of Zagreb showed that school menus do not provide adequate amounts or a sufficient variety of fruits and vegetables [85].

Adolescents often consume less milk and dairy products, which may contribute to the low calcium intake [86]. In our study sample, milk and dairy products account for 11.3% of total food consumed, averaging 247.5 ± 189.8 g per day. Milk and fermented dairy products were the most commonly consumed food items. Although both adolescent age groups consumed the same amount of milk and dairy products, the contribution to daily energy intake was greater in older adolescents, reflecting their slightly higher cheese consumption. These results suggest suboptimal consumption of dairy products. However, Croatian adolescents consumed a similar amount of milk and dairy products compared to their peers in Portugal, Belgium, and the Netherlands [55,63,67], and higher intakes than adolescents in some other European countries [18,65,66]. The consumption of milk and dairy products by children and adolescents has been studied in relation to obesity risk, but there is no clear evidence to support this association [87]. Therefore, adequate consumption of milk and dairy products within national recommendations should be encouraged.

The last food group of particular interest is beverages. This was the most consumed food group among adolescents in Croatia, with higher intake observed in older adolescents. The mean beverage intake was 1212.4 ± 650.5 mL per day. This group included soft drinks, tea with and without sugar, energy drinks, coffee with and without sugar, and fruit nectars. Therefore, it cannot be compared with EFSA recommendations for fluid intake as it accounts for all fluid intake from beverages and food. Despite the overall high consumption of this group in our study population, it contributed only 2.9% to daily energy intake and 3.7% to total carbohydrate intake. This may be explained by the fact that the majority of adolescents consumed water, while only two consumed energy drinks and fruit nectars, and 149 consumed non-alcoholic drinks containing sugars and sweeteners. Additionally, only 39 adolescents consumed tea with added sugar, while all consumed coffee with added sugar. Data from the HELENA study show that, along with high water intake, sugar-sweetened beverages are among the most commonly consumed drinks and contribute to 30.4% of daily energy intake [88]. More recent findings from the HBSC indicate daily consumption of sugar-sweetened soft drinks among approximately 14% of 11-year-olds, 16% of 13-year-olds, and 16% of 15-year-olds, with significant differences between countries and a higher prevalence among boys [54]. Higher consumption of sugar-sweetened beverages is considered a public health concern, mainly due to the high intake of added sugars. Frequent consumption of these beverages has been associated with higher BMI and a higher risk of cardiometabolic diseases [89]. EFSA recommends minimizing added sugar intake by limiting consumption of sugar-sweetened beverages [90]. There is growing emphasis on regulating marketing practices that may influence food choice and increase consumption of sugar-sweetened beverages, especially among children and adolescents [91].

The frequency of meal consumption may influence dietary intake and overall diet quality. Our study results are consistent with existing studies that observe frequent breakfast skipping [6,92]. Skipping breakfast has been associated with adverse weight status [93], cardiometabolic risk factors [94], low academic achievement [95] and mental health [96], which consequently may affect adolescents’ quality of life. Moreover, it has been related to the overall low diet quality [92]. Moderate snacking throughout the day is an integral part of a balanced diet, but we are witnessing a shift toward more frequent small meals replacing traditional eating patterns [97,98]. Opinion on snacking during adolescence is divided. While increasing the frequency of nutrient-dense snacks may contribute to better diet quality, frequent consumption of discrete snacks may reduce diet quality and is often associated with sedentary lifestyles [99,100,101,102,103]. In this study, we used a questionnaire to assess the frequency of consumption of a morning, afternoon, or evening snack. However, these data did not capture the number or nutritional quality of snacks consumed. The results indicate that adolescents consumed snacks more frequently in the afternoon and evening than in the morning, which is in line with previous findings [104]. A potential negative aspect of snack consumption later in the day is shifting energy intake to the evening hours. High evening energy intake has been associated with an increased odds for obesity and a greater likelihood of skipping breakfast or consuming a less appropriate breakfast [105,106]. According to the Croatian physical activity in adolescence longitudinal study (CROPALS study), shifting energy intake to late hours is already present among Croatian adolescents, although its association with nutritional status is limited [28]. Future studies should aim to provide evidence-based guidelines by examining the frequency, timing, and quality of snacking meals per day, as well as their consequences on overall diet quality. It is also important to continue encouraging the habit of eating lunch and dinner at home, as it is associated with better diet quality [27]. Future guidelines and public health campaigns should focus on increasing breakfast consumption among adolescents.

In Croatia, there are currently no FBDG or standardized dietary assessment tools developed for assessing nutritional quality. Therefore, the DQI-A was used because it has been validated in adolescent populations from Austria, Germany, Hungary, and Mediterranean countries such as Italy, Spain, and Greece, supporting its applicability in both continental and coastal regions of Croatia. However, interpretation of these results should consider that the index is based on the Flemish FBGD. Although Flemish dietary guidelines may differ from Croatian dietary habits, dietary recommendations are generally based on principles of healthy nutrition. In the absence of Croatian-specific guidelines, the Flemish framework was considered an appropriate and methodologically robust reference. Within this context, overall diet quality was assessed as moderate among Croatian adolescents using DQI-A. Moderate diet quality has already been observed among adolescents from both the continental and Mediterranean regions of Croatia using the KIDMED index [107]. There are many different indices that can be used to assess overall diet quality, most of which reflect adherence to a specific dietary pattern, such as the Mediterranean diet or national dietary guidelines [108]. In Croatia, there are no FBDGs for adolescents nor a nationally developed diet quality index. Therefore, DQI-A was used to estimate overall diet quality [49]. Adolescents in the HELENA study had higher diet quality than those observed in our study [109]. The same index has been applied in the UK adolescent population, who had lower diet quality (mean: 21.1; 95% CI 20.5–21.8) compared to Croatian adolescents [19]. The Diet Quality Index-International is a version similar to the DQI-A, reflecting variety, adequacy, moderation, and overall balance in the adolescent diet. Using this index, studies in Italy, Portugal, and Spain have observed moderate overall diet quality among adolescents, with 50% or more of the total score achieved [21,22,110]. Low diet quality during adolescence has been linked to health problems, including obesity and reduced quality of life [20,21,22,110]. Several factors may influence adolescents’ diet quality, including age, household income, activity level, sleep duration, meal frequency and quality, and breakfast skipping [19,21,92,110,111,112,113,114]. Future studies examining the determinants of diet quality in the Croatian population are warranted, as they may help in designing targeted public health strategies.

This is the first comprehensive study on adolescent dietary patterns and quality in Croatia. A major strength of this study is the use of a stratified national sample of adolescents in Croatia, which allows generalizing conclusions to the population level. Additionally, the sample was stratified by age, sex, and region, and data were collected to represent all seasons. The study was conducted using the rigorous EU Menu methodology [2], which ensures the quality of the collected data and allows for comparison with other relevant studies in Europe. Despite the proposed methodology, the study has several limitations that should be highlighted. Of the total study sample, only 10 adolescents independently completed the questionnaires and 24 h recalls. For the remaining adolescents, the questionnaires and dietary assessments were completed with parental assistance, which may lead to underestimation of food and beverages consumed outside the home [115]. In Croatia, there are no FBDG or dietary recommendations for evaluating adolescents’ dietary patterns. Therefore, we used EFSA DRV to assess compliance with current recommendations [48] and DQI-A validated in the European adolescent population for diet quality assessment [49]. Analysis included identified under-reporters, which led to underestimation of energy and nutrient intake, as well as some food groups that individuals consider unacceptable for consumption. Since adolescents’ diets vary daily, and this study considered only two 24 h recalls with data analyzed based on their mean value, identifying the prevalence of adolescents who met the EFSA DRV may be subject to bias. Therefore, these findings reflect observed intake and should not be interpreted as precise estimates of usual intake adequacy, but rather as a descriptive indicator of intake. We did not present detailed nutrient intake, such as fatty acid profiles, sugars, and dietary fiber, because we could not adequately assess these parameters due to insufficient data in the chemical composition tables and the inability to substitute certain foods from other sources. Additionally, the chemical composition of food products on the Croatian market is based on nutrition labels, which are required to include only information on energy value, amounts of fat, saturated fat, carbohydrates, sugar, protein, and salt. Given the existence of different versions of the DQI-A for assessing diet quality, data from future studies should be interpreted with caution. Furthermore, data were collected during the COVID-19 pandemic, which may have affected dietary patterns.

## 5. Conclusions

This study demonstrated that adolescents in Croatia have moderate overall diet quality. Additionally, based on the mean of two 24 h recalls, the present findings suggest limited adherence to current European dietary recommendations. Several unfavorable dietary patterns were identified, including insufficient fruit, vegetables, legumes, and milk and dairy products. Meanwhile, the cakes, confectionery, sweets, and sugar food group were among the main contributors to daily energy intake. Analysis of meal frequency reveals that the majority of adolescents skip breakfast and tend to consume more meals in the afternoon and evening. These data suggest a need for the development of evidence-based dietary guidelines for this population group, school food policies that support healthy eating patterns, broader school environment improvements and targeted public health educational campaigns. However, to design targeted activities, future research on the determinants of adolescents’ eating behavior is warranted.

## Figures and Tables

**Table 1:** Socio-demographic and lifestyle characteristics of adolescents from the NIPNOD 2018–2023 survey.

Variables	Total Sample (N = 258)	Age 10–13 (*n* = 123)	Age 14–17 (*n* = 135)	*p* Value *
Sex (%):				
Boys	50.4	56.1	45.2	0.083
Girls	49.6	43.9	54.8	
Age (yr.)	14.3 ± 3.2	12.2 ± 1.1	16.3 ± 1.1	<0.001
Region (%):				
Dalmatia	18.2	21.1	15.6	0.064
Istra, Primorje and Gorski Kotar	12.4	12.2	12.6	
Lika and Banovina	7.8	6.5	8.9	
Northern Croatia	24.8	20.3	28.9	
Slavonia	13.6	19.5	8.10	
Zagreb	23.3	20.3	25.9	
Place of residence (%):				
Urban	76.4	74.4	78.2	0.554
Rural	23.6	25.6	21.8	
Parents’ education level ^1^ (%):				
Low	5.6	4.9	6.4	0.913
Medium	39.1	39.8	38.4	
High	55.2	55.3	55.2	
Parents’ employment status (%):				
Employed for pay or profit	85.9	87.0	84.8	0.825
Student	1.2	0.8	1.6	
Unemployed	8.9	8.1	9.6	
Maternity leave	0.8	1.6	0.0	
Domestic	2.0	1.6	2.4	
Retired	1.2	0.8	1.6	
Household income ^1^ (%):				
<451 €	1.2	0.8	1.5	0.949
451–902 €	5.1	5.8	4.5	
903–1354 €	21.3	20.7	21.8	
1355–1805 €	72.4	72.7	72.2	
Presence of chronic disease (%):				
No	88.0	85.4	90.4	0.252
Yes	12.0	14.6	9.6	
Physical activity level ^2^ (%):				
Low	45.7	48.8	43.0	0.312
Medium	22.9	18.7	26.7	
High	31.4	32.5	30.4	

Continuous data are presented as mean ± standard deviation, and categorical data as percentages. ^1^ Education levels were classified according to the International Standard Classification of Education (ISCED) as low (0–2), medium (3–4), and high (5–8). ^2^ The monetary unit in Croatia was the Croatian kuna (HRK), with an average exchange rate of 1 HRK = 7.53450 EUR. * Differences between age groups were tested using the independent *t*-test for continuous data and the Chi-square test or Fisher’s exact test for categorical data (*p* < 0.05).

**Table 2:** Anthropometric characteristics of adolescents from the NIPNOD 2018–2023 survey.

Variables	Total Sample (N = 258)	Age 10–13 (*n* = 123)	Age 14–17 (*n* = 135)	*p* Value *
Body weight (kg)	56.4 ± 15.4	47.4 ± 10.3	64.6 ± 14.6	<0.001
Body height (cm)	165.0 ± 12.2	157.2 ± 9.4	172.1 ± 9.8	<0.001
z-score of body mass index for age	0.2 ± 1.1	0.3 ± 1.1	0.2 ± 1.1	0.463
Category of z-score of body mass index for age (%):				
Underweight	2.3	2.4	2.2	0.496
Normal weight	71.3	69.1	73.3	
Overweight	21.7	25.2	18.5	
Obese	4.7	3.3	5.9	

Continuous data are presented as mean ± standard deviation, and categorical data as percentages. * Differences between age groups were tested using the independent *t*-test or Mann–Whitney U test for continuous data, and the Chi-square test or Fisher’s exact test for categorical data (*p* < 0.05).

**Table 3:** Mean daily energy and macronutrient intake among adolescents from the NIPNOD 2018–2023 survey.

Nutrient	Mean ± SD	P 5	P 25	P 50	P 75	P 95	*p* Values *
Energy (kcal)							
Total sample (N = 258)	1820 ± 529	1004	1508	1793	2079	2760	
Age 10–13 (*n* = 123)	1758 ± 425	1014	1531	1712	1952	2521	0.125
Age 14–17 (*n* = 135)	1876 ± 605	958	1505	1853	2153	2897	
Carbohydrates (g)							
Total sample (N = 258)	205.9 ± 62.9	103.8	167.9	203.2	235.0	327.8	
Age 10–13 (*n* = 123)	207.1 ± 54.8	116.8	173.4	209.8	231.7	314.0	0.426
Age 14–17 (*n* = 135)	204.8 ± 69.7	92.1	162.4	199.2	239.9	336.2	
Carbohydrates (% kJ)							
Total sample (N = 258)	45.5 ± 7.0	34.9	40.5	45.8	50.0	56.9	
Age 10–13 (*n* = 123)	47.3 ± 6.4	36.6	42.7	47.5	51.6	56.9	<0.001
Age 14–17 (*n* = 135)	43.9 ± 7.1	31.8	39.3	43.5	48.3	57.1	
Protein (g)							
Total sample (N = 258)	68.4 ± 23.7	36.6	52.2	64.6	81.4	107.2	
Age 10–13 (*n* = 123)	64.4 ± 18.5	39.9	51.6	61.0	74.8	103.6	0.021
Age 14–17 (*n* = 135)	72.2 ± 27.0	35.9	52.8	67.8	85.2	116.2	
Protein (g/kg BW)							
Total sample (N = 258)	1.3 ± 0.5	0.6	0.9	1.2	1.6	2.3	
Age 10–13 (*n* = 123)	1.4 ± 0.5	0.7	1.1	1.4	1.8	2.4	<0.001
Age 14–17 (*n* = 135)	1.2 ± 0.5	0.5	0.9	1.1	1.4	2.1	
Protein (% kJ)							
Total sample (N = 258)	15.1 ± 3.1	11.1	12.9	14.7	17.0	20.5	
Age 10–13 (*n* = 123)	14.7 ± 2.5	11.0	12.8	14.3	16.3	19.9	0.074
Age 14–17 (*n* = 135))	15.5 ± 3.4	11.2	13.0	15.1	17.5	21.7	
Fat (g)							
Total sample (N = 258)	77.1 ± 28.5	40.6	57.3	73.3	91.3	128.4	
Age 10–13 (*n* = 123)	71.7 ± 22.9	40.6	55.1	68.9	83.3	114.2	0.006
Age 14–17 (*n* = 135)	82.0 ± 32.0	38.9	60.4	78.2	97.9	135.7	
Fat (% kJ)							
Total sample (N = 258)	37.8 ± 6.3	27.6	33.8	37.7	42.0	47.8	
Age 10–13 (*n* = 123)	36.5 ± 6.1	26.8	32.2	36.5	40.2	46.5	0.001
Age 14–17 (*n* = 135)	39.0 ± 6.1	28.2	35.1	38.5	42.9	49.6	

* Differences between age groups were tested using the independent *t*-test or Mann–Whitney U test (*p* < 0.05).

**Table 4:** Descriptive characteristics of the Diet Quality Index for Adolescents (DQI-A) and its components in adolescents from the NIPNOD 2018–2023 survey.

Variables	Total Sample (N = 258)	Age 10–13 (*n* = 123)	Age 14–17 (*n* = 135)	*p* Value *
DQI-A (%)	57.4 ± 11.6	56.6 ± 11.7	58.1 ± 11.5	0.142
Dietary quality (%)	49.8 ± 25.7	47.3 ± 25.2	52.0 ± 26.2	0.075
Dietary diversity (%)	79.8 ± 12.4	79.1 ± 12.7	80.4 ± 12.1	0.415
Dietary equilibrium (%)	42.6 ± 8.9	43.4 ± 9.2	41.9 ± 8.5	0.194

Data are presented as mean ± standard deviation. * Differences between age groups were tested using the independent *t*-test or the Mann–Whitney U test (*p* < 0.05).

## Data Availability

The data are available from the Croatian Agency for Agriculture and Food, but restrictions apply to the availability of these data. Data are available upon request and with permission of the Croatian Agency for Agriculture and Food.

## Supplementary Materials

The following supporting information can be downloaded at: https://www.mdpi.com/article/10.3390/children13060799/s1, Table S1: List of food categories and subcategories used in NIPNOD 2018–2023 survey; Table S2: Mean daily intake of energy and macronutrients among adolescents by sex in the 10–13 age group from the NIPNOD 2018–2023 survey; Table S3: Mean daily intake of energy and macronutrients among adolescents by sex in the 14–17 age group from the NIPNOD 2018–2023 survey; Table S4: Distribution of adolescents from the NIPNOD 2018–2023 survey by macronutrient intake relative to EFSA DRVs, by sex and age group; Table S5: Mean daily food group intake in adolescents from NIPNOD 2018–2023 survey; Table S6: Mean daily food group intake in adolescents by sex in the 10–13 age group from NIPNOD 2018–2023 survey; Table S7: Mean daily food group intake in adolescents by sex in the 14–17 age group from NIPNOD 2018–2023 survey; Table S8: Relative contribution of food groups to the total daily food consumption in adolescents from NIPNOD 2018–2023 survey; Table S9: Relative contribution of food groups to the total daily energy intake in adolescents from NIPNOD 2018–2023 survey; Table S10: Mean relative contribution of food groups to the daily macronutrient intake in adolescents from NIPNOD 2018–2023 survey; Table S11: Distribution of adolescent from NIPNOD 2018–2023 survey according to the frequency of meals consumption; Table S12: Descriptive characteristics of the Diet Quality Index for Adolescents (DQI-A) and its components in adolescents by sex and age from NIPNOD 2018–2023 survey; Table S13: Post hoc analysis regarding plausibility reporters of mean daily intake of energy and macronutrients among adolescents from the NIPNOD 2018–2023 survey; Table S14: Post hoc analysis regarding plausibility reporters of distribution of adolescent from NIPNOD 2018–2023 survey according to the frequency of meals consumption; Table S15: Post hoc analysis regarding plausibility reporters of average daily food group intake in adolescents from NIPNOD 2018–2023 survey; Table S16: Post hoc analysis regarding plausibility reporters of relative contribution of the food groups to the total daily food consumption in adolescents from NIPNOD 2018–2023 survey; Table S17: Post hoc analysis regarding plausibility reporters of relative contribution of the food groups to the total daily energy intake in adolescents from NIPNOD 2018–2023 survey; Table S18: Post hoc analysis regarding plausibility reporters of distribution of adolescent from NIPNOD 2018–2023 survey according to the frequency of meals consumption; Table S19: Post hoc analysis in plausible adolescent reports from NIPNOD 2018–2023 survey according to the frequency of meals consumption; Table S20: Post hoc analysis regarding plausibility reporters of diet quality of adolescents from NIPNOD 2018–2023 survey.

## Abbreviations

The following abbreviations are used in this manuscript:

BMI	Body mass index
BW	Body weight
DRV	Dietary reference values
DQI-A	Diet quality index for adolescents
EFSA	European Food Safety Authority
FBDG	Food-based dietary guidelines
HBSC	Health Behaviour in School-age Children Study
HELENA	Healthy Lifestyle in Europe by Nutrition in Adolescence Study
KIDMED	Mediterranean diet quality index for children and adolescents
NIPNOD	National food consumption survey on adolescents and adults from 10 to 99 years of age in Croatia
PANCAKE	Pilot study for the Assessment of Nutrient intake and food Consumption Among Kids in Europe
WHO	World Health Organization
