# Supplementary material for: National Food Consumption Survey (NIPNOD 2018–2023): Results of Dietary Habits and Diet Quality Among Adolescents in Croatia
Source: Children (Basel). 2026 Jun 10;13(6):799. doi: 10.3390/children13060799 (PMC13297248; doi:10.3390/children13060799)
Supplement: Supplementary file 1 [file children-13-00799-s001.zip › children-4278956-supplementary.pdf]

Supplementary Materials

Table S1. List of food categories and subcategories used in NIPNOD 2018-2023 survey

Food groups	Food subcategory
Grains, grain products, potatoes and tubers	Bread, rolls and tortillas<br>Grains, grits and flour<br>Pasta<br>Breakfast cereals<br>Fresh and frozen potatoes and tubers<br>Fresh and frozen potatoes and tubers products
Fruit	Fresh, canned, frozen fruits<br>100% fruit juices<br>Dried fruits and fruit bars (% fruit)
Vegetables	Fresh, canned, frozen vegetables<br>100% vegetables juices<br>Dried vegetables
Legumes, seeds and nuts	Fresh, dried, canned and frozen legumes<br>Seeds and seeds products without added salt and sugar<br>Nuts and nuts products without added salt and sugar<br>Milk and meat alternatives
Meat, poultry, fish and eggs	Fresh and frozen read meat and poultry<br>Fresh and frozen fish and seafood<br>Eggs<br>Meat, poultry and fish products (processed, canned or breaded)
Milk and dairy products	Milk<br>Flavoured milk<br>Fermented dairy products<br>Flavoured fermented dairy products<br>Dairy desserts<br>Cheese and cheese spreads
Fats	Plant oils<br>Animal fats
Beverages	Water<br>Tea<br>Fruit and vegetable juices<br>Energy drinks<br>Other non-alcoholic beverages
Alcoholic beverages	Wine<br>Beer<br>Liqueurs<br>Strong alcoholic beverages other than liqueurs<br>Fortified and liqueur wines<br>Other mixed alcoholic beverages<br>Vinegar
Complementary foods	Ready-to-eat baby porridges and desserts made of fruit and vegetables<br>Read–to-eat baby meals<br>Ready-to-eat cereal and milk porridges

Table S1. *conitnued*

Salty snacks	Grain based salty snacks<br>Potato based salty snacks
Cakes, confectionery, sweets and sugar	Chocolate and chocolate spreads with additives<br>Cocoa powder<br>Biscuits and dry cakes<br>Cakes and tarts<br>Marmalades, jams and jellies<br>Honey and sweeteners<br>Ice creams based on water and/or milk<br>Sugar<br>Confectionery and chewing gum<br>Other
Dietetic products	Supplements<br>Enteral supplementation
Miscellaneous foods	Salt<br>Condiment with dehydrate vegetables, spices and salt<br>Yeast<br>Dehydrated stocks

Table S2. Mean daily intake of energy and macronutrients among adolescents by sex in the 10–13 age group from the NIPNOD 2018–2023 survey

Variables	Boys (n = 69)						Girls (n = 54)					
	Mean ± SD	P 5	P 25	P 50	P 75	P 95	Mean ± SD	P 5	P 25	P 50	P 75	P 95
**Energy (kcal)**	1881 ± 453	1148	1598	1841	2194	2743	1601 ± 328	940	1479	1644	1841	2089
**Carbohydrates (g)**	219.8 ± 57.9	119.6	181.9	216.0	256.5	341.6	190.8 ± 46.2	104.7	151.6	199.9	220.3	274.8
**Carbohydrates (% kJ)**	46.8 ± 5.7	36.6	42.5	47.5	50.8	56.6	47.9 ± 7.2	34.6	42.9	47.5	52.3	59.9
**Protein (g)**	68.0 ± 20.1	36.8	53.1	65.8	81.8	105.6	59.7 ± 15.2	37.9	50.3	57.2	67.4	95.9
**Protein (g/kg BW)**	1.5 ± 0.5	0.7	1.2	1.4	1.8	2.4	1.3 ± 0.4	0.7	1.0	1.3	1.6	2.3
**Protein (% kJ)**	14.5 ± 2.6	10.8	12.5	14.1	16.1	19.6	15.0 ± 2.5	11.4	13.0	14.5	16.7	20.0
**Fat (g)**	78.1 ± 22.3	45.8	59.9	76.3	91.4	122.6	63.6 ± 21.2	32.6	49.7	61.3	74.0	110.2
**Fat (% kJ)**	37.3 ± 5.1	28.9	33.5	37.5	40.4	46.4	35.4 ± 7.1	22.6	30.8	34.7	39.8	50.3

Table S3. Mean daily intake of energy and macronutrients among adolescents by sex in the 14–17 age group from the NIPNOD 2018–2023 survey

**Variables**	**Boys (n = 61)**						**Girls (n = 74)**					
	**Mean ± SD**	**P 5**	**P 25**	**P 50**	**P 75**	**P 95**	**Mean ± SD**	**P 5**	**P 25**	**P 50**	**P 75**	**P 95**
**Energy (kcal)**	2132 ± 604	1263	1745	2023	2463	2917	1665 ± 520	838	1363	1650	1973	2774
**Carbohydrates (g)**	230.8 ± 63.1	141.2	194.0	218.1	263.7	347.8	183.3 ± 67.9	79.3	139.8	1771.1	216.0	329.2
**Carbohydrates (% kJ)**	43.8 ± 6.8	32.9	39.2	43.5	47.7	57.8	43.9 ± 7.4	31.4	39.4	43.3	48.5	57.3
**Protein (g)**	85.2 ± 30.2	44.5	66.1	81.0	98.3	148.2	61.4 ± 18.3	34.0	49.2	58.9	76.6	95.1
**Protein (g/kg BW)**	1.3 ± 0.5	0.5	1.0	1.2	1.4	2.3	1.1 ± 0.4	0.4	0.8	1.0	1.4	1.9
**Protein (% kJ)**	16.0 ± 3.4	11.2	13.9	15.5	17.8	22.0	15.1 ± 3.4	11.0	12.8	14.3	17.0	21.4
**Fat (g)**	93.0 ± 35.0	43.3	69.2	89.6	110.6	142.8	73.0 ± 26.4	35.1	52.2	72.5	88.6	122.9
**Fat (% kJ)**	38.7 ± 5.9	27.8	34.7	39.2	42.6	47.7	39.2 ± 6.3	28.0	36.0	38.3	43.4	51.4

Table S4. Distribution of adolescents from the NIPNOD 2018–2023 survey by macronutrient intake relative to EFSA DRVs, by sex and age group

**Nutrient**	**Age 10 – 13**						**Age 14 – 17**					
	**Boys (n = 69)**			**Girls (n = 54)**			**Boys (n = 61)**			**Girls (n = 74)**		
	**Below DRV**	**Adequate intake**	**Above DRV**	**Below DRV**	**Adequate intake**	**Above DRV**	**Below DRV**	**Adequate intake**	**Above DRV**	**Below DRV**	**Adequate intake**	**Above DRV**
**Carbohydrates**	34.8%	65.2%	0.0%	35.2%	61.1%	3.7%	59.0%	39.9%	1.6%	55.4%	41.9%	2.7%
**Protein**	13.0%	0.0%	87.0%	13.0%	3.7%	83.3%	16.4%	4.9%	78.7%	20.3%	12.2%	67.6%
**Fat**	0.0%	34.8%	65.2%	0.0%	50.0%	50.0%	0.0%	26.2%	73.8%	0.0%	21.6%	78.4%

Table S5. Mean daily food group intake in adolescents from NIPNOD 2018-2023 survey

**Food group (g/day)**	**Mean ± SD**	**P 5**	**P 25**	**P 50**	**P 75**	**P 95**	**p Values***
**Grains, grain products, potatoes and tubers**							
Total sample (N = 258)	258.2 ± 100.6	114.5	191.4	247.5	304.7	425.5	
Age 10 – 13 (n = 123)	246.1 ± 88.5	101.9	191.9	243.9	291.1	393.7	0.178
Age 14 – 17 (n = 135)	269.2 ± 109.7	115.8	188.0	251.4	328.5	516.1	
**Fruit**							
Total sample (N = 258)	150.3 ± 151.2	0.0	5.7	111.0	230.6	450.6	
Age 10 – 13 (n = 123)	154.5 ± 145.5	0.0	22.0	128.5	229.0	449.9	0.320
Age 14 – 17 (n = 135)	146.6 ± 156.7	0.0	0.0	99.5	231.0	453.4	
**Vegetables**							
Total sample (N = 258)	108.7 ± 79.6	19.3	51.5	90.1	141.0	275.6	
Age 10 – 13 (n = 123)	105.8 ± 76.8	13.2	50.6	89.8	137.6	168.5	0.681
Age 14 – 17 (n = 135)	111.3 ± 82.3	19.8	52.7	90.5	144.5	294.1	
**Legumes, seeds and nuts**							
Total sample (N = 258)	17.4 ± 40.2	0.0	0.0	0.0	15.4	98.0	
Age 10 – 13 (n = 123)	16.5 ± 37.5	0.0	0.0	0.0	17.1	97.0	0.294
Age 14 – 17 (n = 135)	18.2 ± 42.6	0.0	0.0	0.0	14.9	59.8	
**Meat, poultry, fish and eggs**							
Total sample (N = 258)	152.6 ± 74.7	49.2	10.2.2	141.1	196.0	297.1	
Age 10 – 13 (n = 123)	138.0 ± 66.3	49.3	99.2	123.6	168.5	274.8	0.002
Age 14 – 17 (n = 135)	165.9 ± 79.4	49.0	114.9	160.7	204.5	303.6	
**Milk and dairy products**							
Total sample (N = 258)	247.5 ± 189.8	13.1	119.6	217.7	346.6	541.8	
Age 10 – 13 (n = 123)	265.0 ± 192.5	15.0	125.0	240.0	379.4	580.4	0.095
Age 14 – 17 (n = 135)	231.6 ± 186.5	10.0	111.6	211.6	317.4	536.9	
**Fats**							
Total sample (N = 258)	21.2 ± 12.5	4.2	12.9	18.9	26.9	45.0	
Age 10 – 13 (n = 123)	19.6 ± 10.6	4.3	12.0	28.9	26.4	40.5	0.151
Age 14 – 17 (n = 135)	22.7 ± 13.8	4.1	13.4	18.9	30.8	51.9	

Table S5. *continued*

**Beverages**							
Total sample (N = 258)	1212.4 ± 650.5	397.6	139.9	1048.7	1520.7	2459.7	
Age 10 – 13 (n = 123)	1086.7 ± 547.6	389.2	675.6	949.0	1438.9	2145.4	0.006
Age 14 – 17 (n = 135)	1327.0 ± 714.7	441.0	833.9	1233.5	1621.5	2724.8	
**Alcoholic beverages**							
Total sample (N = 258)	5.5 ± 32.4	0.0	0.0	0.0	2.3	8.9	
Age 10 – 13 (n = 123)	1.3 ± 2.2	0.0	0.0	0.0	2.1	6.0	0.391
Age 14 – 17 (n = 135)	9.2 ± 47.0	0.0	0.0	0.0	2.3	24.5	
**Complementary foods**							
Total sample (N = 258)	0.7 ± 11.8	0.0	0.0	0.0	0.0	0.0	
Age 10 – 13 (n = 123)	0.0 ± 0.0	0.0	0.0	0.0	0.0	0.0	0.340
Age 14 – 17 (n = 135)	1.4 ± 16.4	0.0	0.0	0.0	0.0	0.0	
**Salty snacks**							
Total sample (N = 258)	6.5 ± 17.2	0.0	0.0	0.0	0.0	45.1	
Age 10 – 13 (n = 123)	8.0 ± 17.3	0.0	0.0	0.0	0.0	50.0	0.041
Age 14 – 17 (n = 135)	5.2 ± 17.1	0.0	0.0	0.0	0.0	42-0	
**Cakes, confectionery, sweets and sugar**							
Total sample (N = 258)	83.0 ± 79.6	0.0	22.2	60.0	115.9	242.8	
Age 10 – 13 (n = 123)	89.1 ± 77.4	0.2	31.3	70.0	126.0	225.7	0.058
Age 14 – 17 (n = 135)	77.5 ± 81.5	0.0	12.0	52.0	109.5	260.1	
**Dietetic products**							
Total sample (N = 258)	3.4 ± 28.1	0.0	0.0	0.0	0.0	6.2	
Age 10 – 13 (n = 123)	5.5 ± 40.2	0.0	0.0	0.0	0.0	5.8	0.299
Age 14 – 17 (n = 135)	1.4 ± 5.9	0.0	0.0	0.0	0.0	0.6	
**Miscellaneous foods**							
Total sample (N = 258)	4.3 ± 4.8	0.9	2.3	3.4	5.1	8.8	
Age 10 – 13 (n = 123)	4.0 ± 3.2	0.7	2.2	3.5	5.1	8.6	0.939
Age 14 – 17 (n = 135)	4.6 ± 5.8	1.1	2.3	3.4	4.8	11.8	

*Differences between age groups were tested using the independent t-test or Mann–Whitney U test ($p < 0.05$).

Table S6. Mean daily food group intake in adolescents by sex in the 10–13 age group from NIPNOD 2018-2023 survey

**Food group (g/day)**	**Boys (n = 69)**						**Girls (n = 54)**					
	**Mean ± SD**	**P 5**	**P 25**	**P 50**	**P 75**	**P 95**	**Mean ± SD**	**P 5**	**P 25**	**P 50**	**P 75**	**P 95**
Grains, grain products, potatoes and tubers	251. ± 89.5	82.4	182.7	257.9	311.5	391.8	239.5 ± 87.6	98.7	202.6	234.8	257.8	447.5
Fruit	156.8 ± 147.6	0.0	12.0	122.5	227.9	467.4	151.3 ± 144.1	0.0	35.5	138.9	230.4	358.8
Vegetables	105.7 ± 76.8	11.1	47.3	89.3	154.9	264.4	105.9 ± 77.5	15.9	54.6	90.8	130.6	289.3
Legumes, seeds and nuts	21.5 ± 46.5	0.0	0.0	0.3	22.0	135.4	10.0 ± 19.7	0.0	0.0	0.0	14.9	51.5
Meat, poultry, fish and eggs	150.7 ± 68.9	52.3	103.8	136.9	183.3	300.1	121.8 ± 59.7	44.3	83.0	105.7	155.5	241.6
Milk and dairy products	271.3 ± 211.0	10.9	121.4	245.8	392.8	612.6	257.0 ± 167.5	37.2	131.5	223.1	337.5	615.5
Fats	21.4 ± 11.4	3.6	12.0	20.3	30.0	42.2	17.2 ± 9.3	4.8	10.2	17.5	21.0	35.4
Beverages	1113.8 ± 555.8	374.1	732.6	982.2	1453.6	2184.7	1051.9 ± 540.1	408.2	648.5	899.7	1409.7	2205.1
Alcoholic beverages	1.3 ± 2.2	0.0	0.0	0.0	2.4	6.6	1.4 ± 2.2	0.0	0.0	0.0	2.1	6.0
Complementary foods	0.0 ± 0.0	0.0	0.0	0.0	0.0	0.0	0.0 ± 0.0	0.0	0.0	0.0	0.0	0.0
Salty snacks	9.2 ± 19.7	0.0	0.0	0.0	4.2	49.1	6.3 ± 13.6	0.0	0.0	0.0	0.0	41.0
Cakes, confectionery, sweets and sugar	94.3 ± 73.1	0.0	34.5	84.3	135.1	231.3	82.4 ± 82.8	0.0	26.2	58.5	106.0	227.5
Dietetic products	6.4 ± 48.1	0.0	0.0	0.0	0.0	7.0	4.3 ± 27.3	0.0	0.0	0.0	0.0	7.6
Miscellaneous foods	4.1 ± 2.2	0.9	2.5	3.7	5.7	8.4	4.0 ± 4.2	0.6	1.9	3.4	4.8	9.7

Table S7. Mean daily food group intake in adolescents by sex in the 14–17 age group from NIPNOD 2018-2023 survey

**Food group (g/day)**	**Boys (n = 61)**						**Girls (n = 74)**					
	**Mean ± SD**	**P 5**	**P 25**	**P 50**	**P 75**	**P 95**	**Mean ± SD**	**P 5**	**P 25**	**P 50**	**P 75**	**P 95**
Grains, grain products, potatoes and tubers	312.8 ± 103.4	142.9	243.1	301.1	374.4	523.4	233.3 ± 102.0	98.2	168.5	219.7	274.3	435.2
Fruit	156.3 ± 172.3	0.0	0.0	99.5	291.8	453.8	138.5 ± 143.4	0.0	7.1	93.8	202.4	457.3
Vegetables	128.0 ± 96.2	20.6	62.3	94.0	186.0	365.4	97.5 ± 66.3	19.3	48.8	86.1	123.7	229.5
Legumes, seeds and nuts	13.3 ± 30.1	0.0	0.0	0.0	5.2	92.3	22.2 ± 50.5	0.0	0.0	0.0	18.8	174.9
Meat, poultry, fish and eggs	201.6 ± 82.7	62.9	142.3	195.2	246.2	340.3	136.4 ± 63.4	39.7	91.2	130.7	175.9	271.5
Milk and dairy products	282.6 ± 226.6	13.3	144.0	240.0	367.5	778.7	189.5 ± 133.1	8.3	93.6	177.8	275.4	436.9
Fats	26.1 ± 15.4	6.9	15.2	22.4	38.1	57.1	19.9 ± 11.8	3.9	13.0	17.4	25.0	42.5
Beverages	1404.6 ± 854.4	333.7	843.3	12500	1779.7	3307.8	1263.1 ± 573.1	483.8	822.1	1224.4	1589.5	2329.5
Alcoholic beverages	9.5 ± 45.1	0.0	0.0	0.0	2.1	9.8	9.0 ± 48.8	0.0	0.0	0.9	2.7	26.9
Complementary foods	0.0 ± 0.0	0.0	0.0	0.0	0.0	0.0	2.6 ± 22.1	0.0	0.0	0.0	0.0	0.0
Salty snacks	5.4 ± 14.9	0.0	0.0	0.0	0.0	44.6	5.1 ± 18.9	0.0	0.0	0.0	0.0	39.6
Cakes, confectionery, sweets and sugar	79.6 ± 89.8	0.0	9.2	51.8	114.0	287.8	75.7 ± 74.6	0.0	12.0	52.1	110.9	245.0
Dietetic products	2.7 ± 8.4	0.0	0.0	0.0	0.0	30.0	0.3 ± 1.7	0.0	0.0	0.0	0.0	1.6
Miscellaneous foods	5.4 ± 7.4	1.3	2.8	3.8	5.3	15.9	3.9 ± 4.9	0.0	2.0	3.0	4.7	8.6

Table S8. Relative contribution of food groups to the total daily food consumption in adolescents from NIPNOD 2018-2023 survey

**Food group (% g)**	**Total sample (N = 258)**	**Age 10 – 13 (n = 123)**			**Age 14 – 17 (n = 135)**			**p Values***
		**Total (n=123)**	**Boys (n=69)**	**Girls (n=54)**	**Total (n=123)**	**Boys (n=61)**	**Girls (n=74)**	
Grains, grain products, potatoes and tubers	12.2%	12.3%	12.1%	12.5%	12.1%	13.1%	11.2%	0.567
Fruit	6.7%	7.0%	6.9%	7.2%	6.4%	6.4%	6.4%	0.116
Vegetables	5.0%	5.1%	5.0%	5.1%	4.9%	5.3%	4.6%	0.568
Legumes, seeds and nuts	0.8%	0.8%	1.1%	0.5%	0.8%	0.5%	1.0%	0.275
Meat, poultry, fish and eggs	7.1%	6.7%	7.0%	6.3%	7.4%	8.3%	6.7%	0.215
Milk and dairy products	11.3%	12.9%	12.8%	13.1%	9.8%	10.9%	8,8%	0.006
Fats	1.0%	0.9%	1.0%	0.9%	1.0%	1.1%	1.0%	0.619
Beverages	51.1%	48.9%	48.5%	49.4%	53.1%	50.1%	55.6%	0.011
Alcoholic beverages	0.2%	0.1%	0.1%	0.1%	0.4%	0.3%	0.4%	0.452
Complementary foods	0.0%	0.0%	0.0%	0.0%	0.0%	0.0%	0.1%	0.340
Salty snacks	0.3%	0.4%	0.4%	0.4%	0.2%	0.2%	0.2%	0.034
Cakes, confectionery, sweets and sugar	3.9%	4.3%	4.4%	4.1%	3.5%	3.3%	3.7%	0.005
Dietetic products	0.2%	0.3%	0.4%	0.2%	0.1%	0.1%	0.0	0.299
Miscellaneous foods	0.2%	0.2%	0.2%	0.2%	0.2%	0.3%	0.2%	0.285

*Differences between age groups were tested using the independent t-test or Mann–Whitney U test ($p < 0.05$).

Table S9. Relative contribution of food groups to the total daily energy intake in adolescents from NIPNOD 2018-2023 survey

**Food group (% g)**	**Total sample (N = 258)**	**Age 10 – 13 (n = 123)**			**Age 14 – 17 (n = 135)**			**p Values***
		**Total (n=123)**	**Boys (n=69)**	**Girls (n=54)**	**Total (n=123)**	**Boys (n=61)**	**Girls (n=74)**	
Grains, grain products, potatoes and tubers	31.5%	31.1%	29.4%	33.2%	31.9%	32.6%	31.3%	0.705
Fruit	5.0%	5.1%	4.5%	5.8%	5.0%	4.6%	5.3%	0.377
Vegetables	2.4%	2.4%	2.4%	2.4%	2.4%	2.6%	2.4%	0.834
Legumes, seeds and nuts	1.4%	1.4%	1.7%	1.1%	1.4%	1.1%	1.7%	0.262
Meat, poultry, fish and eggs	18.1%	17.1%	5.0%	4.7%	19.1%	6.5%	5.3%	0.048
Milk and dairy products	11.2%	11.2%	10.8%	11.8%	11.2%	11.1%	11.2%	0.994
Fats	10.0%	9.5%	9.8%	9.2%	10.4%	10.8%	10.1%	0.244
Beverages	2.9%	2.9%	3.4%	2.2%	3.0%	3.2%	2.8%	0.412
Alcoholic beverages	0.1%	0.0%	0.0%	0.0%	0.2%	0.1%	0.3%	0.418
Complementary foods	0.0%	0.0%	0.0%	0.0%	0.0%	0.0%	0.0%	0.340
Salty snacks	1.8%	2.3%	2.4%	2.1%	1.3%	1.4%	1.3%	0.034
Cakes, confectionery, sweets and sugar	14.9%	16.2%	16.9%	15.3%	13.6%	11.7%	15.2%	0.020
Dietetic products	0.4%	0.6%	0.9%	0.3%	0.2%	0.4%	0.1%	0.581
Miscellaneous foods	0.2%	0.1%	0.1%	0.2%	0.2%	0.1%	0.2%	0.115

*Differences between age groups were tested using the independent t-test or Mann–Whitney U test ($p < 0.05$).

Table S10. Mean relative contribution of food groups to the daily macronutrient intake in adolescents from NIPNOD 2018-2023 survey

Food group	Carbohydrates (%)	Protein (%)	Fat (%)
**Grains, grain products, potatoes and tubers**			
Total sample (N = 258)	53.8	23.4	8.8
Age 10 – 13 (n = 123)	51.5	23.6	8.2
Boys (n = 69)	49.6	23.0	7.1
Girls (n = 54)	53.8	22.8	8.4
Age 14 – 17 (n = 135)	55.9	23.2	9.3
Boys (n = 61)	53.6	24.4	9.7
Girls (n = 74)	54.1	23.5	10.1
**Fruit**			
Total sample (N = 258)	9.4	1.5	0.3
Age 10 – 13 (n = 123)	9.3	1.5	0.3
Boys (n = 69)	8.4	1.4	0.2
Girls (n = 54)	10.3	1.5	0.3
Age 14 – 17 (n = 135)	9.5	1.5	0.3
Boys (n = 61)	10.3	1.6	0.4
Girls (n = 74)	10.2	1.5	0.4
**Vegetables**			
Total sample (N = 258)	3.4	2.6	0.8
Age 10 – 13 (n = 123)	3.4	2.6	0.6
Boys (n = 69)	3.5	2.6	0.6
Girls (n = 54)	3.2	2.7	0.9
Age 14 – 17 (n = 135)	3.5	2.6	0.9
Boys (n = 61)	3.2	2.7	0.7
Girls (n = 74)	3.4	2.4	0.9
**Legumes, seeds and nuts**			
Total sample (N = 258)	1.0	1.6	1.7
Age 10 – 13 (n = 123)	0.9	1.8	1.8
Boys (n = 69)	1.1	2.1	2.3
Girls (n = 54)	0.6	1.0	1.2
Age 14 – 17 (n = 135)	1.1	1.0	1.5
Boys (n = 61)	0.6	1.4	1.3
Girls (n = 74)	1.5	2.0	1.8
**Meat, poultry, fish and eggs**			
Total sample (N = 258)	0.6	42.7	28.8
Age 10 – 13 (n = 123)	0.7	41.5	27.8
Boys (n = 69)	0.9	42.7	28.3
Girls (n = 54)	0.4	45.1	32.7
Age 14 – 17 (n = 135)	0.5	43.8	29.7
Boys (n = 61)	0.4	40.0	27.2
Girls (n = 74)	0.5	42.8	27.2

Table S10. *conitnued*

**Milk and dairy products**			
Total sample (N = 258)	6.7	18.9	14.3
Age 10 – 13 (n = 123)	7.3	19.5	13.5
Boys (n = 69)	7.4	18.3	12.4
Girls (n = 54)	7.2	17.8	14.6
Age 14 – 17 (n = 135)	6.0	18.4	15.0
Boys (n = 61)	7.2	21.0	14.8
Girls (n = 74)	5.8	18.8	15.3
**Fats**			
Total sample (N = 258)	0.0	0.1	26.4
Age 10 – 13 (n = 123)	0.0	0.1	26.2
Boys (n = 69)	0.0	0.1	26.1
Girls (n = 54)	0.0	0.1	27.3
Age 14 – 17 (n = 135)	0.0	0.1	26.7
Boys (n = 61)	0.0	0.1	26.2
Girls (n = 74)	0.0	0.1	26.1
**Beverages**			
Total sample (N = 258)	5.7	0.5	0.3
Age 10 – 13 (n = 123)	5.7	0.1	0.1
Boys (n = 69)	6.9	0.0	0.1
Girls (n = 54)	4.3	0.5	0.4
Age 14 – 17 (n = 135)	5.7	0.8	0.5
Boys (n = 61)	4.3	0.1	0.1
Girls (n = 74)	5.0	1.1	0.7
**Alcoholic beverages**			
Total sample (N = 258)	0.1	0.0	0.0
Age 10 – 13 (n = 123)	0.0	0.0	0.0
Boys (n = 69)	0.0	0.0	0.0
Girls (n = 54)	0.0	0.0	0.0
Age 14 – 17 (n = 135)	0.2	0.0	0.0
Boys (n = 61)	0.0	0.0	0.0
Girls (n = 74)	0.2	0.0	0.0
**Complementary foods**			
Total sample (N = 258)	0.0	0.0	0.0
Age 10 – 13 (n = 123)	0.0	0.0	0.0
Boys (n = 69)	0.0	1.0	0.0
Girls (n = 54)	0.0	0.0	0.0
Age 14 – 17 (n = 135)	0.0	0.0	0.0
Boys (n = 61)	0.0	0.0	0.0
Girls (n = 74)	0.2	0.0	0.0
**Salty snacks**			
Total sample (N = 258)	1.6	0.8	2.5
Age 10 – 13 (n = 123)	2.1	1.0	3.2
Boys (n = 69)	2.3	0.0	3.2
Girls (n = 54)	1.8	0.5	2.0
Age 14 – 17 (n = 135)	1.1	0.6	1.9
Boys (n = 61)	1.8	0.9	3.2
Girls (n = 74)	2.2	0.6	1.8

Table S10. *conitnued*

**Cakes, confectionery, sweets and sugar**			
Total sample (N = 258)	17.2	7.1	15.5
Age 10 – 13 (n = 123)	18.3	7.7	17.4
Boys (n = 69)	18.7	8.0	18.6
Girls (n = 54)	17.8	6.2	11.8
Age 14 – 17 (n = 135)	16.1	6.5	13.9
Boys (n = 61)	17.8	7.4	15.8
Girls (n = 74)	17.9	6.7	15.6
**Dietetic products**			
Total sample (N = 258)	0.3	0.7	0.4
Age 10 – 13 (n = 123)	0.7	0.5	0.7
Boys (n = 69)	0.9	0.6	0.9
Girls (n = 54)	0.3	1.7	0.1
Age 14 – 17 (n = 135)	0.0	0.9	0.1
Boys (n = 61)	0.3	0.2	0.4
Girls (n = 74)	0.0	0.2	0.0
**Miscellaneous foods**			
Total sample (N = 258)	0.1	0.2	0.2
Age 10 – 13 (n = 123)	0.1	0.1	0.2
Boys (n = 69)	0.1	0.1	0.2
Girls (n = 54)	0.1	0.2	0.2
Age 14 – 17 (n = 135)	0.1	0.2	0.2
Boys (n = 61)	0.1	0.2	0.3
Girls (n = 74)	0.2	0.3	0.2

Table S11. Distribution of adolescent from NIPNOD 2018-2023 survey according to the frequency of meals consumption.

Meal	Age group	Never	1 per week	2 per week	3 per week	4 per week	5 per week	6 per week	Every day	p Value
Breakfast	Total sample (N = 258)	41.2	1.6	7.0	7.0	4.7	9.7	1.9	26.8	
	Age 10 - 13 (n = 123)	36.9	0.8	2.5	6.6	8.2	12.3	1.6	31.1	0.043
	Age 14 - 17 (n = 135)	45.2	2.2	11.1	7.4	1.5	7.4	2.2	23.0	
Morning snack	Total sample (N = 258)	68.5	3.5	0.4	1.9	6.6	4.7	8.9	5.4	
	Age 10 - 13 (n = 123)	72.1	3.3	0.0	0.8	4.9	5.7	8.2	4.9	0.741
	Age 14 - 17 (n = 135)	65.2	3.7	0.7	3.0	8.1	3.7	9.6	5.9	
Lunch	Total sample (N = 258)	0.0	0.0	0.0	0.4	1.2	0.8	1.6	96.1	
	Age 10 - 13 (n = 123)	0.0	0.0	0.0	0.8	0.0	0.8	0.0	98.4	0.041
	Age 14 - 17 (n = 135)	0.0	0.0	0.0	0.0	2.2	0.7	3.0	94.1	
Afternoon snack	Total sample (N = 258)	3.9	0.8	4.7	8.6	9.0	19.1	3.9	50.0	
	Age 10 - 13 (n = 123)	0.8	0.0	0.8	8.2	6.6	26.2	3.3	54.1	0.001
	Age 14 - 17 (n = 135)	6.7	1.5	8.2	9.0	11.2	12.7	4.5	46.3	
Dinner	Total sample (N = 258)	0.4	0.0	0.4	2.3	3.1	2.7	3.9	87.2	
	Age 10 - 13 (n = 123)	0.0	0.0	0.0	0.0	2.5	0.8	4.1	92.6	0.042
	Age 14 - 17 (n = 135)	0.7	0.0	0.7	4.4	3.7	4.4	3.7	82.2	
Evening snack	Total sample (N = 258)	27.5	7.5	19.2	12.9	8.2	9.4	2.0	13.3	
	Age 10 - 13 (n = 123)	20.5	8.2	23.0	12.3	11.5	8.2	2.5	13.9	0.194
	Age 14 - 17 (n = 135)	33.8	6.8	15.8	13.5	5.3	10.5	1.5	12.8	

* Differences between age groups were tested using the Chi-square or Fisher's exact test ($p < 0.05$).

Table S12. Descriptive characteristics of the Diet Quality Index for Adolescents (DQI-A) and its components in adolescents by sex and age from NIPNOD 2018-2023 survey

Variables	Age 10 – 13		Age 14 – 17	
	Boys (n=69)	Girls (n=54)	Boys (n=61)	Girls (n=74)
**DQI-A (%)**	54.7 ± 11.3	59.1 ± 11.8	58.3 ± 12.3	57. ± 10.8
**Dietary quality (%)**	43.3 ± 25.7	52.5 ± 23.8	49.7 ± 26.2	53.8 ± 26.2
**Dietary diversity (%)**	78.9 ± 11.3	79.4 ± 14.4	83.2 ± 12.1	78.1 ± 11.6
**Dietary equilibrium (%)**	41.8 ± 8.8	45.4 ± 9.4	42.0 ± 8.5	41.9 ± 8.6

Table S13. Post hoc analysis regarding plausibility reporters of mean daily intake of energy and macronutrients among adolescents from the NIPNOD 2018–2023 survey regarding energy plausibility

Nutrient	Total study sample			Total plausible reports		
	Total misreports (n = 84)	Total plausible reports (n = 174)	p Values*	Age 10 – 13 (n = 92)	Age 14 – 17 (n = 82)	p Values**
**Energy (kcal)**	1405 ± 404	2020 ± 462	< 0.001	1917 ± 345	2136 ± 554	0.006
**Carbohydrates (g)**	157.0 ± 47.1	229.5 ± 55.6	< 0.001	225.8 ± 46.4	233.6 ± 64.5	0.795
**Carbohydrates (% kJ)**	44.9 ± 21.1	45.7 ± 6.9	0.382	47.3 ± 6.5	43.9 ± 7.0	0.001
**Protein (g)**	55.6 ± 20.3	74.6 ± 22.7	< 0.001	69.5 ± 17.0	80.3 ± 26.8	0.004
**Protein (g/kg BW)**	0.9 ± 0.3	1.5 ± 0.5	< 0.001	1.6 ± 0.4	1.4 ± 0.5	< 0.001
**Protein (% kJ)**	15.9 ± 3.2	14.8 ± 2.9	0.005	14.5 ± 2.5	15.1 ± 3.3	0.308
**Fat (g)**	58.9 ± 21.1	85.9 ± 27.4	< 0.001	78.5 ± 21.5	94.2 ± 30.9	< 0.001
**Fat (% kJ)**	37.5 ± 5.9	38.0 ± 6.4	0.551	36.6 ± 6.3	39.5 ± 6.3	0.003

The variables are presented as mean ± SD. Differences were tested using the independent t-test or Mann–Whitney U test (* between misreports and plausible reports; ** between age groups of plausible reports; $p < 0.05$).

Table S14. Post hoc analysis of the plausibility of reports of distribution of adolescent from NIPNOD 2018-2023 survey according to the frequency of meals consumption

Nutrient	**Total study sample**							**Total plausible reports**						
	**Total misreports (n = 84)**			**Total plausible reports (n = 174)**			p Value*	**Age 10 – 13 (n = 92)**			**Age 14 – 17 (n = 82)**			p Value**
	Below DRV	Adequate intake	Above DRV	Below DRV	Adequate intake	Above DRV		Below DRV	Adequate intake	Above DRV	Below DRV	Adequate intake	Above DRV	
Carbohydrates	50.0%	47.6%	2.4%	44.8%	53.4%	1.7%	0.662	33.7%	64.1%	2.2%	57.3%	41.5%	1.2%	0.007
Protein	44.0%	9.5%	46.4%	2.3%	3.4%	94.3%	< 0.001	0.0%	2.2%	97.8%	4.9%	4.9%	90.2%	0.050
Fat	0.0%	32.1%	67.9%	0.0%	32.2%	67.8%	0.990	0.0%	41.3%	58.7%	0.0%	22.0%	78.0%	0.006

Differences were tested using the Chi-square or Fisher’s exact test (* between misreports and plausible reports; ** between age groups of plausible reports; p < 0.05).

Table S15. Post hoc analysis of the plausibility of reports of average daily food group intake in adolescents from NIPNOD 2018-2023 survey

**Food group (g/day)**	**Total study sample**			**Total plausible reports**		
	**Total misreports (n = 84)**	**Total plausible reports (n = 174)**	**p Values***	**Age 10 – 13 (n = 92)**	**Age 14 – 17 (n = 82)**	**p Values****
Grains, grain products, potatoes and tubers	214.7 ± 97.2	279.2 ± 95.6	< 0.001	264.6 ± 83.0	295.5 ± 106.2	0.031
Fruit	160.1 ± 143.0	145.5 ± 155.2	0.239	161.6 ± 153.3	127.6 ± 156.3	0.040
Vegetables	99.6 ± 75.5	113.1 ± 81.3	0.116	107.1 ± 79.7	110.7 ± 83.1	0.227
Legumes, seeds and nuts	15.3 ± 36.2	18.4 ± 42.0	0.340	16.5 ± 35.0	20.6 ± 48.9	0.281
Meat, poultry, fish and eggs	137.2 ± 69.1	160.1 ± 76.3	0.025	143.2 ± 68.2	178.9 ± 80.7	< 0.001
Milk and dairy products	178.9 ± 148.3	280.6 ± 198.9	< 0.001	306.6 ± 68.2	251.5 ± 200.6	0.016
Fats	17.1 ± 10.8	23.2 ± 12.8	< 0.001	21.4 ± 10.6	25.2 ± 14.7	0.227
Beverages	1342.0 ± 779.4	1149.9 ± 570.1	0.167	1079.5 ± 524.0	1228.8 ± 611.4	0.100
Alcoholic beverages	2.0 ± 4.7	7.1 ± 41.5	0.851	1.3 ± 2.2	13.7 ± 59.9	0.128
Complementary foods	0.0. ± 0.0	1.1 ± 14.4	0.487	0.0 ± 0.0	2.3 ± 21.0	0.289
Salty snacks	3.4 ± 10.0	8.0 ± 19.6	0.108	9.2 ± 18.9	6.7 ± 20.4	0.085
Cakes, confectionery, sweets and sugar	48.8 ± 52.4	99.5 ± 85.2	< 0.001	100.2 ± 82.2	98.8 ± 88.9	0.660
Dietetic products	5.8 ± 43.8	2.2 ± 15.8	0.683	2.9 ± 20.9	1.4 ± 6.19	0.437
Miscellaneous foods	4.0 ± 6.2	4.5 ± 3.9	0.007	4.3 ± 3.5	4.7 ± 4.3	0.714

The variables are presented as mean ± SD. Differences were tested using the independent t-test or Mann–Whitney U test (* between misreports and plausible reports; ** between age groups of plausible reports; $p < 0.05$).

Table S16. Post hoc analysis of the plausibility of reports of relative contribution of the food groups to the total daily food consumption in adolescents from NIPNOD 2018-2023 survey

**Food group (% g)**	**Total study sample**			**Total plausible reports**		
	**Total misreports (n = 84)**	**Total plausible reports (n = 174)**	**p Values***	**Age 10 – 13 (n = 92)**	**Age 14 – 17 (n = 82)**	**p Values****
Grains, grain products, potatoes and tubers	10.6%	13.0%	0.001	12.7%	13.3%	0.583
Fruit	7.7%	6.2%	0.141	7.1%	5.2%	0.013
Vegetables	4.9%	5.0%	0.310	4.9%	5.2%	0.507
Legumes, seeds and nuts	0.8%	0.8%	0.339	0.8%	0.9%	0.270
Meat, poultry, fish and eggs	6.5%	7.3%	0.187	6.7%	8.1%	0.015
Milk and dairy products	8.4%	12.7%	< 0.001	14.5%	10.6%	0.002
Fats	0.8%	1.1%	0.002	1.0%	1.2%	0.392
Beverages	56.0%	48.3%	< 0.001	46.8%	49.9%	0.072
Alcoholic beverages	0.1%	0.3%	0.931	0.1%	0.6%	0.121
Complementary foods	0.0%	0.0%	0.487	0.0%	0.1%	0.289
Salty snacks	0.2%	0.4%	0.134	0.4%	0.3%	0.076
Cakes, confectionery, sweets and sugar	2.5%	4.6%	< 0.001	4.7%	4.4%	0.315
Dietetic products	0.4%	0.1%	0.679	0.1%	0.1%	0.433
Miscellaneous foods	0.2%	0.2%	0.020	0.2%	0.2%	0.878

The variables are presented as mean ± SD. Differences were tested using the independent t-test or Mann–Whitney U test (* between misreports and plausible reports; ** between age groups of plausible reports; $p < 0.05$).

Table S17. Post hoc analysis of the plausibility of reports of relative contribution of the food groups to the total daily energy intake in adolescents from NIPNOD 2018-2023 survey

**Food group (% kcal)**	**Total study sample**			**Total plausible reports**		
	**Total misreports (n = 84)**	**Total plausible reports (n = 174)**	**p Values***	**Age 10 – 13 (n = 92)**	**Age 14 – 17 (n = 82)**	**p Values****
Grains, grain products, potatoes and tubers	31.2%	31.7%	0.983	31.4%	32.0%	0.835
Fruit	6.9%	4.1%	< 0.001	4.7%	3.5%	0.022
Vegetables	2.8%	2.3%	0.035	2.2%	2.3%	0.664
Legumes, seeds and nuts	1.6%	1.3%	0.480	1.4%	1.2%	0.216
Meat, poultry, fish and eggs	20.4%	17.0%	0.003	15.9%	18.2%	0.026
Milk and dairy products	10.8%	11.4%	0.375	11.8%	10.9%	0.298
Fats	10.2%	9.9%	0.330	9.6%	10.2%	0.828
Beverages	2.7%	3.0%	0.253	2.8%	3.3%	0.195
Alcoholic beverages	0.1%	0.2%	0.611	0.0%	0.3%	0.140
Complementary foods	0.0%	0.0%	0.487	0.0%	0.0%	0.289
Salty snacks	1.3%	2.0%	0.152	2.5%	1.5%	0.072
Cakes, confectionery, sweets and sugar	11.0%	16.7%	< 0.001	17.3%	16.1%	0.433
Dietetic products	0.8%	0.2%		0.2%	0.2%	0.783
Miscellaneous foods	0.2%	0.1%	0.900	0.1%	0.2%	0.084

The variables are presented as mean ± SD. Differences were tested using the independent t-test or Mann–Whitney U test (* between misreports and plausible reports; ** between age groups of plausible reports; $p < 0.05$).

Table S18. Post hoc analysis of the plausibility of reports of distribution of adolescent from NIPNOD 2018-2023 survey according to the frequency of meals consumption

Meals		**Never**	**1 per week**	**2 per week**	**3 per week**	**4 per week**	**5 per week**	**6 per week**	**Every day**	**p Value***
Breakfast	Misreports (n = 84)	44.0	1.2	8.3	11.9	3.6	8.3	2.4	20.2	0.363
	Plausible reports (n = 174)	39.9	1.7	6.4	4.6	5.2	10.4	1.7	30.1	
Morning snack	Misreports (n = 84)	63.1	4.8	0.0	3.6	8.3	7.1	10.7	2.4	0.328
	Plausible reports (n = 174)	71.1	2.9	0.6	1.2	5.8	3.5	8.1	6.9	
Lunch	Misreports (n = 84)	0.0	0.0	0.0	0.0	3.6	0.0	0.0	96.4	0.048
	Plausible reports (n = 174)	0.0	0.0	0.0	0.6	0.0	1.2	2.3	96.0	
Afternoon snack	Misreports (n = 84)	4.8	2.4	8.4	14.5	13.3	18.1	1.2	37.3	0.003
	Plausible reports (n = 174)	3.5	0.0	2.9	5.8	6.9	19.7	5.2	56.1	
Dinner	Misreports (n = 84)	0.0	0.0	1.2	1.2	3.6	3.6	3.6	86.9	0.718
	Plausible reports (n = 174)	0.6	0.0	0.0	2.9	2.9	2.3	4.0	87.3	
Evening snack	Misreports (n = 84)	34.9	8.4	16.9	14.5	8.4	9.6	1.2	6.0	0.287
	Plausible reports (n = 174)	23.8	7.0	20.3	12.2	8.1	9.3	2.3	16.9	

Differences were tested using Chi-square/Fisher’s exact test (* $p < 0.05$).

Table S19. Post hoc analysis in plausible adolescent reports from NIPNOD 2018-2023 survey according to the frequency of meals consumption

Meals	Age group	Never	1 per week	2 per week	3 per week	4 per week	5 per week	6 per week	Every day	p Value
**Breakfast**	Age 10 – 13 (n = 92)	33.0	1.1	2.2	3.3	8.8	13.2	2.2	36.3	0.014
	Age 14 – 17 (n = 82)	47.6	2.4	11.0	6.1	1.2	7.3	1.2	23.2	
**Morning snack**	Age 10 – 13 (n = 92)	72.5	4.4	0.0	0.0	4.4	4.4	7.7	6.6	0.531
	Age 14 – 17 (n = 82)	69.5	1.2	1.2	2.4	7.3	2.4	8.5	7.3	
**Lunch**	Age 10 – 13 (n = 92)	0.0	0.0	0.0	1.1	0.0	1.1	0.0	97.8	0.144
	Age 14 – 17 (n = 82)	0.0	0.0	0.0	0.0	0.0	1.2	4.9	93.9	
**Afternoon snack**	Age 10 – 13 (n = 92)	1.1	0.0	0.0	4.4	3.3	26.4	4.4	60.4	0.006
	Age 14 – 17 (n = 82)	6.1	0.0	6.1	7.3	11.0	12.2	6.1	51.2	
**Dinner**	Age 10 – 13 (n = 92)	0.0	0.0	0.0	0.0	2.2	0.0	4.4	93.4	0.031
	Age 14 – 17 (n = 82)	1.2	0.0	0.0	6.1	3.7	4.9	3.7	80.5	
**Evening snack**	Age 10 – 13 (n = 92)	20.9	6.6	22.0	11.0	12.1	8.8	3.3	15.4	0.538
	Age 14 – 17 (n = 82)	27.2	7.4	18.5	13.6	3.7	9.9	1.2	18.5	

Differences were tested using the Chi-square or Fisher’s exact test ($p < 0.05$).

Table S20. Post hoc analysis regarding plausibility reporters of diet quality of adolescents from NIPNOD 2018-2023 survey

Variables	Total study sample			Total plausible reports		
	Total misreports (n = 84)	Total plausible reports (n = 174)	p Values*	Age 10 – 13 (n = 92)	Age 14 – 17 (n = 82)	p Values**
**DQI-A (%)**	58.7 ± 13.2	56.8 ± 10.7	0.058	57.5 ± 10.5	56.0 ± 10.9	0.583
**Dietary quality (%)**	58.7 ± 27.3	45.4 ± 23.8	< 0.001	46.1 ± 23.6	44.6 ± 24.2	0.821
**Dietary diversity (%)**	75.5 ± 14.5	81.9 ± 10.6	0.001	81.8 ± 10.5	82.0 ± 10.9	0.775
**Dietary equilibrium (%)**	42.8 ± 8.0	43.0 ± 9.3	0.219	44.5 ± 9.2	41.4 ± 9.2	0.025

The variables are presented as mean ± SD. Differences were tested using the independent t-test or Mann–Whitney U test (* between misreports and plausible reports; ** between age groups of plausible reports; $p < 0.05$).
